# Recent advances in Ti_3_C_2_T_*x*_-based electrolytes for battery applications

**DOI:** 10.1039/d5na00853k

**Published:** 2025-12-19

**Authors:** Ngan Pham Tran Trieu, Vo Thi Thuy Linh, Nguyen Ngoc Tri, Van Nam Huynh, Nguyen Tien Hoang, Qui Thanh Hoai Ta, Soonmin Seo

**Affiliations:** a College of BioNano Technology, Gachon University Gyeonggi 13120 Republic of Korea nganpham1307@gmail.com soonmseo@gachon.ac.kr; b Faculty of Natural Science Education, Pham Van Dong University Quang Ngai Vietnam vttlinh@pdu.edu.vn; c Lab of Computational Chemistry and Modelling, Faculty of Natural Sciences, Quy Nhon University Gia Lai Vietnam nguyenngoctri@qnu.edu.vn; d Faculty of Natural Sciences, Quy Nhon University Gia Lai Vietnam huynhvannam@qnu.edu.vn; e The University of Danang, University of Science and Education 459 Ton Duc Thang st., Lien Chieu Da Nang 550000 Vietnam nthoang@ued.udn.vn; f Institute of Advanced Technology, Vietnam Academy of Science and Technology 1B TL29 Street, An Phu Dong Ward Ho Chi Minh City Vietnam tathanhhoaiqui2292@gmail.com

## Abstract

Since their discovery, two-dimensional Ti_3_C_2_T_*x*_ nanosheets have attracted significant interest for applications in energy storage, including batteries. Among the various strategies developed to enhance their properties, material combination and hybridization have emerged as particularly promising approaches. While much of the current research has centered on the use of Ti_3_C_2_T_*x*_ MXenes in anode or cathode electrode technologies, there is growing interest in exploring single- and multilayer MXenes for electrolyte applications. This expanding scope forms the basis and motivation for the present study.

## Abstract 2

Since their discovery, two-dimensional Ti_3_C_2_T_*x*_ nanosheets have attracted significant interest for applications in energy storage, including batteries.

## Graphical abstract

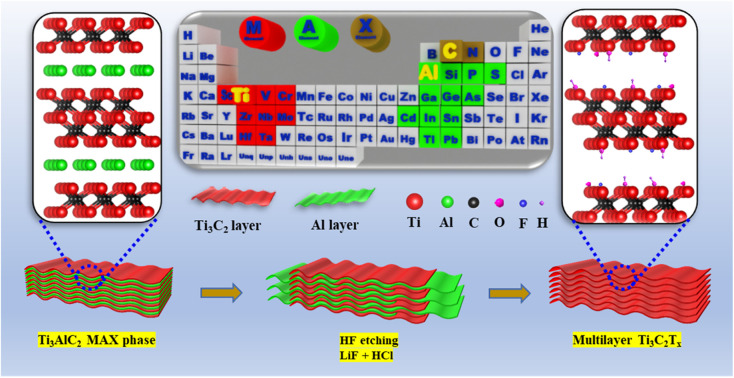

## 1. Introduction

Recently, increasing research attention has been directed toward addressing environmental pollution and the global energy crisis.^1,2^ Owing to its unique advantages, electrochemical energy conversion has emerged as a promising route for green energy production and sustainable development, with a particular focus on the development of rechargeable batteries. In addition to the continuous progress in cathode and anode materials, electrolytes have also been extensively investigated due to their crucial role in governing the electrochemical performance. A properly designed electrolyte that is compatible with electrode reactions can significantly enhance safety, reversibility, and rate capability through improved thermodynamic stability.^3–5^

The Ti_3_C_2_T_*x*_ MXene is one of the most prominent currently known two-dimensional (2D) materials owing to its unique properties, including rapid ion diffusion and the ability to form multilayered structures.^6–8^ MXene synthesis can obtain early transition metal carbides, nitrides, and carbonitrides based on the general formula M_*n*+1_X_*n*_T_*x*_, where M is an early transition metal, X is carbon and/or nitrogen, and T_*x*_ refers to the surface termination groups (*e.g.*, –F, –OH, and –O).^9,10^ The development of Ti_3_C_2_T_*x*_ MXene began in 2011, when Naguib *et al.* first synthesized MXenes through selective etching of the Al layer from the MAX phase.^11^ This seminal study marked a significant milestone in MXene research and led to the synthesis of more than 30 MXene variants with diverse compositions. These materials exhibit distinctive physicochemical properties critical for a wide range of multifunctional applications, prompting further exploration of their use in nanoscience and nanotechnology. With the growing number of investigations on MXenes, researchers now have a broader range of material options that can be tailored to meet specific performance requirements. MXenes exhibit several outstanding properties including multilayered structures with large surface areas, abundant surface functional groups, excellent electronic conductivity, and high mechanical strength. Structurally, Ti_3_C_2_T_*x*_ MXenes possess an accordion-like morphology and, similar to graphite, are held together by weak van der Waals forces. These weak interactions enable the formation of well-defined multilayer structures that provide mechanical stability and a high specific surface area for electron trapping or molecular intercalation. The interlayer spacing and pore gaps offer additional active sites, thereby enhancing adsorption and improving the performance of energy-storage and energy-harvesting applications. Moreover, the presence of covalent, hydrogen, and ionic bonds within the layered framework facilitates efficient charge transfer. Weak van der Waals bonding also enables the exfoliation of MXenes into single- or few-layered nanosheets, further enhancing their physicochemical properties and overall electrochemical performance. Ti_3_C_2_T_*x*_ MXenes possess abundant surface terminations and are intrinsically hydrophilic, facilitating their dispersion in water and various organic solvents. These characteristics improve the interfacial interactions between components and help suppress electrolyte crystallinity in MXene-based electrolyte systems.^12^

Various methods have been developed to synthesize MXenes from MAX phases, including the use of HCl/LiF, HF, HI, and molten salts.^13–15^ However, strong interlayer interactions pose a significant challenge for obtaining high-quality single-layer Ti_3_C_2_T_*x*_ nanosheets in high yields. Various etching and exfoliation techniques have been explored to overcome this limitation. Mathis *et al.* employed a mixed solution of HF/HCl and LiC followed by repeated centrifugation to prepare single-layer Ti_3_C_2_T_*x*_, and this method has been widely adopted for exfoliation of high-quality MXenes.^16,17^ Similarly, Yuchen *et al.* used HF in combination with DMSO to achieve monolayer exfoliation.^18^ Unfortunately, fluorine-containing etchants have several notable drawbacks. In addition to posing significant safety hazards owing to the toxicity of HF, these etchants often result in relatively low yields and may adversely affect the structural integrity of MXene nanosheets. Therefore, the development of safer and more scalable etching methods remains a critical priority for widespread application of MXenes. By introducing Ti_3_C_2_T_*x*_ MXene into ZnSO_4_ aqueous electrolytes, significantly enhanced electrochemical performance has been achieved, including high coulombic efficiency (99.7%) and long-term cycling stability of Zn anodes (1180 cycles). MXenes serve as effective facilitators of rapid charge transport in aqueous electrolytes. Moreover, the presence of MXenes enables more uniform zinc deposition and suppresses dendritic nucleation by shortening the Zn^2+^ diffusion pathways, in clear contrast to electrolytes without MXenes. MXenes have also demonstrated a strong ability to enhance the ion mobility and transport in MXene-based electrolytes. In particular, 3D MXene frameworks facilitate lithium distribution, maintaining a Li-loading efficiency of approximately 92%, even at high current densities, while effectively suppressing dendrite formation for over 2700 h at 0.5 mAh cm^−2^. The high coulombic efficiency (99%) is primarily attributed to the 3D MXene-based electrolyte, which promotes homogeneous lithium nucleation and mitigates dendritic growth. Moreover, Ti_3_C_2_T_*x*_ MXene flakes are favorable substrates for Li deposition because of their large surface areas, excellent conductivities, and intrinsic lithiophilicities. These characteristics give rise to the formation of bowl-shaped Li deposits and prevent excessive volume expansion during repeated electrochemical cycling.^19^

Consequently, considerable research efforts have been devoted to the development of MXene-dispersed electrolytes for advanced energy storage systems, including MXene-based deep eutectic solvent electrolytes (MXene + choline chloride–urea), gel/polymer electrolytes (MXene–PVA–KOH),^20^ ionic liquid electrolytes (MXene–[EMIM][TFSI] and [BMIM][PF_6_]),^21–23^ organic electrolytes (MXene–LiPF_6_ and LiTFSI),^24–26^ and aqueous electrolytes (MXene + KOH, LiOH, or NaOH).^27–29^ Deep eutectic solvents (DESs) have attracted increasing attention owing to their excellent chemical stability, low cost, and inherent safety. These advantages make them promising candidates for synthesis of MXenes with enhanced capacitive performance. Siqui and colleagues reported that MXenes produced *via* a DES-assisted method exhibited abundant –O terminations with a low degree of oxidation, resulting in a high specific capacitance of 320 F g^−1^ at 2 mV s^−1^.^29^ Moreover, under long-term cycling at a current density of 50 A g^−1^, the material maintained 97% of its initial capacitance after 50 000 cycles. This outstanding durability is attributed to the strong interactions between the hydronium ions and –O surface terminations, which promote pseudocapacitive behavior. In addition, MXenes have emerged as key components of gel-type electrolytes for flexible devices, offering high ionic conductivities and self-healing capabilities. Chun *et al.* demonstrated improved performance of zinc-ion batteries by incorporating MXenes into a gel electrolyte composed of polyvinyl alcohol, agar, sodium dodecyl sulfate, and dimethyl sulfoxide. In this system, MXenes enhance ionic conductivity (51 mS cm^−1^) and facilitate uniform Zn^2+^ redistribution, promoting oriented Zn (002) deposition. The optimized MXene-based gel electrolyte enabled a specific capacity of approximately 205 mAh g^−1^, and maintained stable performance for approximately 1000 h at 0.2 A g^−1^ even at a low temperature of −20 °C, while preventing dendrite formation on the anode.^30^

Despite the remarkable progress in the utilization of Ti_3_C_2_T_*x*_ MXenes in energy-storage applications, achieving an optimal balance between electrochemical performance and mechanical robustness, particularly for flexible devices operating at high current densities, remains challenging. Key limitations include the restacking of the Ti_3_C_2_T_*x*_ MXene layers and persistent oxidation resistance. In addition, the porosity of the MXene-based structures must be carefully optimized. A high porosity can lead to a reduced volumetric energy density because the interflake spacing becomes largely filled with the electrolyte, which is neither efficient nor economical for practical device fabrication. Although several reviews on electrolytes have been published, significant gaps remain, and further studies are required to highlight the most recent achievements and address the current challenges in electrolyte technologies.

In this review, we summarize the latest achievements in the functionalization of Ti_3_C_2_T_*x*_ MXenes as electrolytes for energy storage applications. Our discussion begins with an introduction to MXenes, surveying the strategies for their synthesis and their unique physicochemical properties. Subsequently, recent advances in the development of MXene-based systems for energy-harvesting applications are reviewed. Finally, the existing challenges and limitations that must be addressed prior to scaling up MXenes for widespread use in practical applications are discussed.

## 2. Methods for synthesis of Ti_3_C_2_T_*x*_

Typically, layer A in the MAX phase can be selectively removed without breaking the metal–carbon/nitrogen (M–X) bonds. This is due to the weaker chemical bonding between the M and A elements compared to that of the M–X bonds, as well as the influence of the lone-pair electrons on the outer metal atoms, which facilitates the etching process.^31,32^ Multilayered MXene structures are obtained after etching, often requiring intercalation or exfoliation to produce single-layer MXene nanosheets.^10^ Generally, multilayered MXenes can be synthesized *via* top-down selective etching or bottom-up approaches such as chemical vapor deposition.

**Fig. 1:**
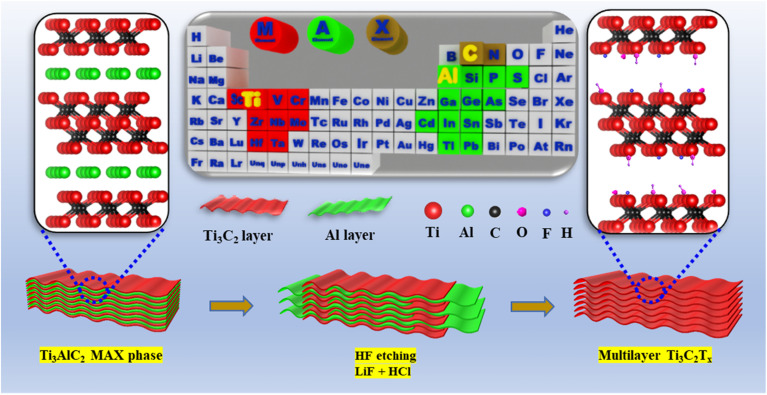
Schematic illustration of the synthesis process of MXenes using a fluorine-based acid.^6^ This figure has been adapted from ref. 6 with permission from Elsevier, copyright 2021.

Since Gogotsi and co-workers first demonstrated that Al layers can be selectively removed from MAX phases using HF to produce MXenes, researchers have expanded and refined MXene synthesis strategies.^11^ For instance, Tran *et al.* successfully synthesized multilayered MXenes by etching the Ti_3_AlC_2_ MAX phase with 50% HF solution (Fig. 1). Scanning electron microscopy images confirmed the structural evolution from the tightly packed bulk Ti_3_AlC_2_ to the characteristic accordion-like morphology of MXenes following the removal of the Al layer.^7^

Although HF is inherently corrosive and poses significant environmental and safety risks, it remains one of the most widely used and effective etchants for the removal of Al layers from MAX phases. Alternative approaches have been developed to mitigate the hazards associated with direct HF usage. For example, a combination of hydrochloric acid (HCl) and lithium fluoride (LiF) can generate HF *in situ* during Ti_3_AlC_2_ etching, offering a safer and more controllable method. This modified route not only reduces handling risks but also broadens the etching strategy, enabling synthesis of novel MXenes with tunable surface termination groups.^33^

The optimal conditions for *in situ* HF generation vary depending on the fluoride salt used. Specifically, etching with NaF/HCl and KF/HCl has been typically conducted at 40 °C for 2 d, while etching at 50 °C for 2 d has been found to be optimal for the LiF/HCl method. The *in situ* HF approach is considered to be easier to implement, more environmentally friendly, and less hazardous than direct HF etching.^34–37^ However, MXenes synthesized *via* direct HF treatment generally exhibit a higher density of F surface functional groups than those synthesized *via in situ* HF etching. This difference in the surface chemistry significantly influences the electrochemical performance of the resulting MXenes.

**Fig. 2:**
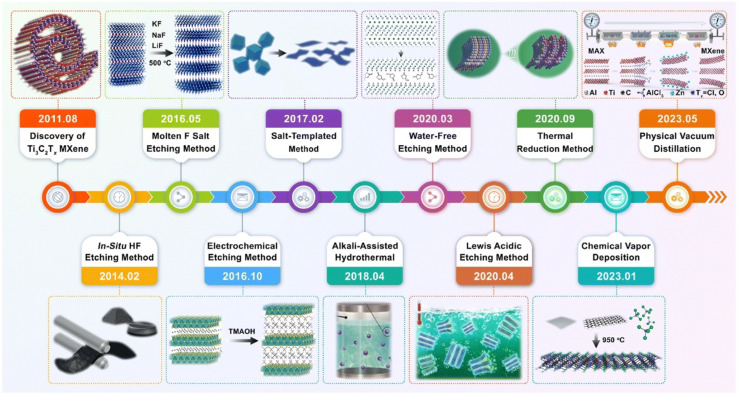
The progress on Ti_3_C_2_T_*x*_ MXene preparation since its first discovery in 2011.^41^ This figure has been adapted from ref. 41 with permission from The Royal Society of Chemistry, copyright 2023.

The most common techniques for synthesis of MXenes involve the use of corrosive acid solutions, such as HF or HCl/LiF. However, these methods generate hazardous waste, posing significant health risks to researchers and contributing to environmental pollution. Moreover, the pH and electrical conductivity of the electrolyte critically influence its compatibility with electrochemical applications. As shown in Table 1, it is imperative to develop alternative synthesis approaches that can produce MXenes with high yield and superior quality, while enabling large-scale, environmentally sustainable production (Fig. 2).^38–40^

**Table 1:** Summary of the advantages and disadvantages associated with various MXene preparation strategies

Technique	Advantages	Disadvantages
Acid etching	A commonly used technique	Requires direct interaction with hazardous HF solution
	Involves complete etching of the MAX phase to obtain MXenes	The etching process is strongly dependent on multiple parameters
		MXenes with many defects
*In situ* acid etching	Enables control over the surface functional groups	The *in situ* generated HF remains toxic
	Avoids direct contact with toxic solutions	Requires extended time to carefully control the reaction
Hydrothermal etching	Does not require the use of highly concentrated acids	The Al layer may not be completely etched
	Facilitates delamination, enabling the preparation of mono- or few-layer MXenes	Reaction parameters must be carefully optimized
Electrochemical etching	Can be scaled up for larger production	Requires sophisticated equipment
	Does not require direct exposure to toxic acids	Often results in non-uniform MXene products
Chemical vapour deposition	Does not require MAX phase precursors	Requires high temperature and pressure conditions
	Can be easily scaled up to pilot-scale production	Control over stoichiometry and impurities is complex and challenging

Overall, the choice of the synthesis strategy depends on the advantages and limitations associated with each method and the requirements of the target application. Although direct HF etching is a simple and versatile route for producing Ti_3_C_2_T_*x*_ MXenes, it often results in materials with high defect densities, presents significant challenges for large-scale manufacturing, and requires the use of toxic reagents. Moreover, this method generates a substantial amount of chemical waste, making recycling difficult and limiting its potential for sustainable applications. By contrast, *in situ* HF generation (*e.g.*, using HCl/LiF) offers a safer and more environmentally friendly alternative. This approach typically yields MXenes with a well-expanded interlayer spacing and a greater number of accessible active sites, contributing to an enhanced specific capacitance. For example, MXenes produced *via* direct HF etching exhibit a lattice parameter of approximately 20 Å, whereas those synthesized using HCl/LiF solutions can reach interlayer spacings of approximately 40 Å. Importantly, *in situ* HF etching is considered to be a milder synthesis route that can produce MXenes with high yields (approaching 99%), large flake sizes, and reduced defect densities; these characteristics are comparable or in some cases even superior to those achieved through direct HF etching. Additionally, the presence of cations in the etching environment further contributes to interlayer expansion, increasing surface accessibility and enhancing adsorption behavior.^42^

Delamination is a crucial step in MXene synthesis, as it enables the conversion of multilayer Ti_3_C_2_T_*x*_ structures into single- or few-layer nanosheets. The interlayer bonding in Ti_3_C_2_T_*x*_ is stronger than that in graphene or MoS_2_. Therefore, additional energy or chemical assistance is required to separate the layers. Density functional theory (DFT) calculations indicated that hydroxylated environments facilitate delamination and that the exfoliation energy for –O terminations is higher than that for –OH terminations, highlighting the influence of surface chemistry on delamination efficiency. Mechanical delamination alone is insufficient for obtaining high-quality monolayer MXenes because it typically requires processing in organic solvents combined with sonication or mechanical vibration. Common intercalation agents such as dimethyl sulfoxide (DMSO) and tetrabutylammonium hydroxide (TBAOH) are widely used to weaken the interlayer interactions. These agents promote delamination and also can modify surface terminations, often reducing the amount of –F groups while increasing the amount of –OH groups. As reported by Thakur *et al.*,^43^ delamination conditions strongly influence flake integrity. Delamination conducted at room temperature without inert-gas protection results in defective and fragmented flakes (∼2.5 µm), and similar degradation occurs at elevated temperatures (*e.g.*, 65 °C). These observations underscore the importance of controlling the temperature and maintaining an inert atmosphere to preserve flake quality during delamination. Soft delamination using a LiCl solution has been shown to produce ultralarge MXene flakes (40 µm), significantly enhancing the mechanical integrity and electrical conductivity of MXene-based electrolytes.

## 3. Properties of Ti_3_C_2_T_*x*_

Ti_3_C_2_T_*x*_ MXene show an exceptional combination of metallic electronic conductivity (up to ∼20 000 S cm^−1^), hydrophilic and highly tunable surface terminations (–O, –OH, and –F), large and adjustable interlayer spacing, and outstanding mechanical stiffness (Young's modulus ∼0.33 TPa). These properties are uniquely suited to electrolyte rather than electrode applications.^44,45^

The most critical attribute for electrolyte design is the dual conductivity of MXenes, which is one of the most electronically conductive 2D materials known and can facilitate efficient ion transport in appropriately designed and engineered electrolyte architectures. The multilayer structure of MXenes provides a large surface area, offering abundant active sites and enhanced ion transport capability. The interlayer spacing can be tuned through surface termination groups and etching techniques, enabling optimization of electrochemical parameters such as ion diffusion pathways.^46^ MXenes exhibit outstanding mechanical properties in electrolyte systems. These mechanical and transport properties are directly exploited to stabilize the interfaces and enable rapid homogeneous ion migration. Surface terminations on Ti_3_C_2_T_*x*_ MXene (–F, –O, –OH) play a decisive role in controlling ion transport kinetics and interfacial chemistry.^32,44,47^ These electronegative groups strongly attract metal cations (Li^+^, Zn^2+^, and Na^+^), enabling MXenes to act as a “cation pump” that significantly lowers desolvation energy barriers, generates cation-rich space-charge layers, and can reach cation transference numbers (*t*^+^) >0.7–0.92 in MXene-based nanochannels and composite membranes while reducing the activation energy for ion migration from ∼0.5 eV (conventional systems) to 0.2–0.4 eV.^32,44,47^ Specifically, –O and –OH terminations provide abundant lithiophilic/zincophilic sites that facilitate rapid cation transport along the MXene surface, promote dissociation from the coordinating anions (*e.g.*, TFSI^−^ and PF_6_^−^), and ensure uniform ion flux with reduced concentration gradients as confirmed by molecular dynamics simulations showing contact angles of ∼30° and enhanced hydration.^44,48,49^ In parallel, tunable interlayer spacing (*via* surface functionalization or cation intercalation) optimizes ion diffusion pathways and prevents restacking more effectively than in inert graphene.^44,50–52^ For high-voltage stability and interface control, –F terminations are uniquely valuable as they serve as an *in situ* fluorine source for forming robust, LiF-rich solid electrolyte interphase (SEI) layers that are mechanically strong, electronically insulating, and ionically conductive. This homogenizes Li^+^ flux, suppresses side reactions, and supports stable operation at elevated voltages (up to ∼4.5–5.0 V).^49,53,54^ However, excess F can reduce the conductivity if it is not balanced by O/OH.

Compared with other 2D materials, Ti_3_C_2_T_*x*_ MXene has two intrinsic advantages for electrolyte design: it is naturally hydrophilic and its surface chemistry is easy to tune.^50,51^ This is particularly evident in the comparison of MXenes to graphene oxide (GO), which can improve ionic conductivity *via* ion-transport pathways but is electrically insulating and cannot provide electron-conductive bridges for current homogenization in contrast to the highly metallic MXene (conductivity of up to 10^4^ S cm^−1^). Similarly, MoS_2_ offers layered mechanics but fewer polar sites for ion affinity, and h-BN stiffens membranes without termination-enabled anion exclusion or SEI control, making MXenes superior for high-rate stable systems.^50–52,55^ In contrast to conventional electrolyte components such as liquid salts or polymer matrices alone, MXene integration enhances safety by reducing flammability and leakage risks, while providing mechanical reinforcement and better interfacial contact, leading to lower resistance (*e.g.*, <100 Ω cm^2^ under pressure) and extended battery lifespan.^56–58^ For instance, compared to typical ceramic solid-state electrolytes (SSEs) with high interfacial resistances (hundreds to thousands of Ω cm^2^) and narrow voltage windows due to poor wettability, the use of MXenes reduces the interfacial resistance to <100 Ω cm^2^ by improving ion flux and SEI stability. The hydrophilic surface terminations and tunable interlayer spacing of MXenes suppress parasitic reactions (*e.g.*, hydrogen evolution), expanding the electrochemical window beyond the ∼1.23 V limit for water to 1.4–3 V in aqueous/Zn systems, outperforming commercial liquid electrolytes with respect to both safety and efficiency.^59–61^

Although heteroatom doping (N, P, and S) and exotic terminations (–Cl, –Br, –S, and –Te) have been widely investigated, primarily in the context of electrocatalysis, their actual impact on MXene-based electrolyte performance remains relatively modest and is often secondary to the effects of native –O/–F/–OH terminations and interlayer spacing control.^62^ Nitrogen doping typically introduces pyridinic and pyrrolic configurations that increase the local electron density, raise the Fermi level, and improve the overall conductivity while lowering activation barriers for ion transfer,^63,64^ analogous to the N-doped graphene aerogels that achieve 98–99% efficiency in energy-related processes owing to better charge separation.^65,66^ Doped phosphorus acts predominantly as an electron donor, generating mid-gap states, increasing the proportion of high-valence Ti^4+^ species, expanding interlayer spacing, and improving hydrophilicity, all of which enhance ion accessibility and active-site exposure.^67–69^ Sulfur doping modulates surface acidity/basicity, boosts polarity, and enhances cation-binding strength, thereby accelerating ion transport and improving cycling stability.^70–72^ Despite these advantages, excessive heteroatom incorporation frequently compromises colloidal stability in polar solvents and accelerates oxidative degradation, which are both critical issues in practical electrolyte systems. In practice, while N/P/S doping can provide measurable gains in specific cases, the dominant performance drivers in real battery electrolytes are still the precisely controlled native –O/–F/–OH ratio and the interlayer spacing. Therefore, heteroatom doping should be considered as a fine-tuning tool rather than the primary strategy for development of MXene-based electrolytes.

**Fig. 3:**
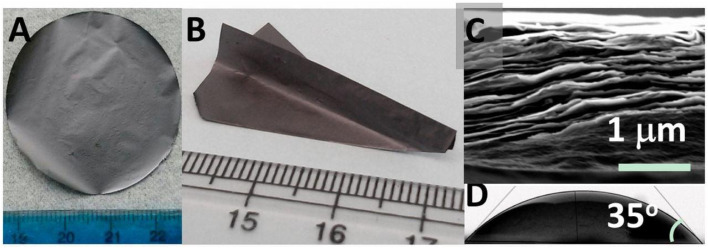
(A) Macroscopic photograph (*D* = 4 cm), (B) the mechanical flexibility, (C) the cross-sectional morphology, and (D) surface wettability of MXenes.^76^ This figure has been reproduced from ref. 76 with permission from National Academy of Sciences, copyright 2014.

The mechanical properties of MXenes are primarily governed by the strengths of M–C and M–N bonds. This structural reinforcement enables MXenes to act as a physical barrier against metal dendrites (Fig. 3).^73–75^ When dispersed in polymer electrolytes, rigid 2D MXene sheets enhance the shear modulus of the matrix, effectively blocking the penetration of sharp Li or Zn filaments (dendrites) and preventing the formation of short circuits.

Although MXenes possess unique physicochemical properties, several challenges must be addressed before their large-scale application. The surface termination groups of MXenes are highly sensitive and prone to oxidation under high-humidity conditions, leading to the loss of metal elements and the creation of TiO_2_ which significantly influence the MXene electrical behavior and electrochemical performance. Therefore, strategies such as surface passivation and deposition of protective layers should be investigated to mitigate oxidation.^77,78^

MXenes possess a large surface area, and their inherent accordion-like morphology can limit ion diffusion between layers. This is a critical drawback for applications such as supercapacitors and batteries, which require rapid ion transport under high-rate charge/discharge conditions. Therefore, it is essential to develop strategies to overcome this limitation, such as construction of porous frameworks, integration of MXenes with highly conductive materials, and precise control of the interlayer spacing. Furthermore, while high electronic conductivity is beneficial for electrodes, it poses a challenge for electrolytes. If the MXene content exceeds the percolation threshold in a solid electrolyte, it can create an electron-conducting network, leading to high self-discharge. Therefore, precise control of the loading mass and surface passivation is required. To address these issues, enhancing the mechanical strength and structural stability of MXenes through composite formation, surface modification, and advanced preparation techniques is of paramount importance.

## 4. Engineered Ti_3_C_2_T_*x*_ MXene-based electrolyte systems

Since its discovery in 2011 by Naguib *et al.*,^11^ Ti_3_C_2_T_*x*_ MXene has attracted attention as a promising material for advanced electrolyte systems due to its exceptional electrical conductivity, large surface area, unique layered structure, and flexible surface chemistry with –OH, –O, and –F groups. These properties enable MXenes to enhance the performance of advanced batteries. However, pure MXenes face issues such as layer restacking, low electrochemical efficiency, and a narrow voltage window, which require engineering solutions to improve their potential.^79^ Beyond fundamental interest, MXene-based electrolytes have practical applications in next-generation systems (*e.g.*, thin, roll-to-roll membranes for EV solid-state packs and moisture-tolerant gels for flexible Zn–air microbatteries), where interface robustness, safety, and manufacturability are vital.

The electrolyte plays a critical role in batteries by ensuring efficient ion transport and charge balance between the electrodes, directly affecting the capacity, charging speed, safety, and practical applications. Electrolytes can be classified into two main types: liquid electrolytes and (quasi)-solid-state electrolytes, which include polymer-based and inorganic electrolytes. Battery performance depends heavily on the active electrochemical sites at the electrode–electrolyte interface; therefore, interface engineering is crucial for boosting efficiency and maintaining mechanical flexibility.^80^ However, inconsistencies in the contact between solid-state electrolytes and electrodes, often due to poor wettability, improper microstructures, or stress cracking, can reduce the effective contact area and adversely affect battery performance. Typical polymer SSEs exhibit initial interfacial resistances of hundreds to thousands of Ω cm^2^ in many solid polymer electrolytes (SPEs) and ceramic systems (*vs.* <100 Ω cm^2^ for liquids) that further increase without stack pressure; therefore, MXene-enabled interphases should be benchmarked under identical pressures to avoid overestimating gains.

**Fig. 4:**
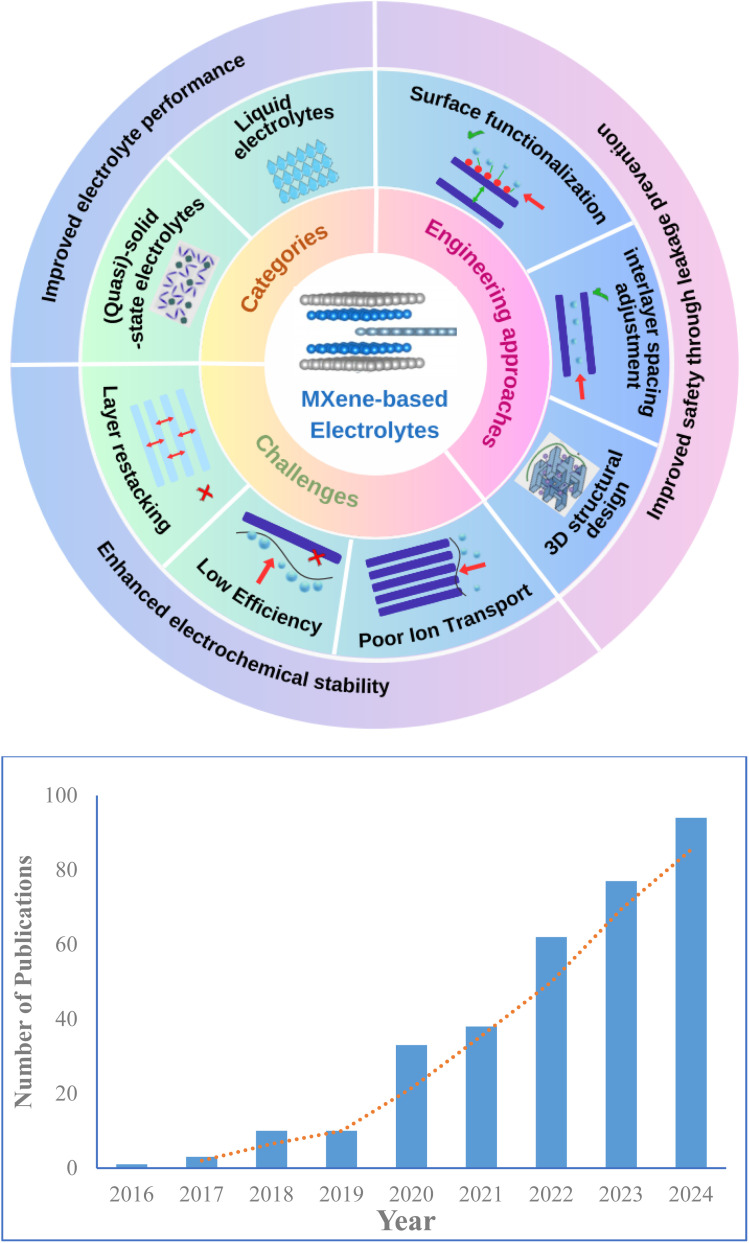
Overview of MXene-based electrolyte systems and total number of publications related to MXene-based electrolytes used in battery/source: Scopus/.

The interaction between the MXene and the electrolyte, particularly through its surface functional groups, plays a significant role in the optimization of the electrode–electrolyte interface.^79^ Engineering approaches such as surface functionalization, heteroatom doping, interlayer spacing adjustments, and 3D structural designs have been used to overcome the limitations of MXenes, expanding their use in high-capacity, safe, and flexible battery systems. These approaches enhance electrolyte performance for properties ranging from ion transport to mechanical flexibility. Fig. 4 shows the trend of research studies on MXene-based electrolytes from 2016 to 2024, showing a sharp rise in publications and highlighting the strong potential of MXenes for next-generation energy-storage applications.

### 4.1. Liquid electrolytes

Liquid electrolyte is the ion-conducting liquid component between the two electrodes in a battery that plays a key role in the transport of ions (such as Li^+^, Na^+^, and Zn^2+^) for the generation of an electric current and in determining the battery's safety and performance. Common liquid electrolytes consist of a solvent (*e.g.*, water, organic compound, or ionic liquid) and a dissolved salt (*e.g.*, Na_2_SO_4_, LiPF_6_, or EMI-TFSI) which dissociates to enable charge transfer.^81^ Each electrolyte type presents a distinct trade-off: aqueous electrolytes offer high ionic conductivity but are limited by a small voltage window; organic electrolytes provide a wider voltage window but are flammable and less conductive; and ionic liquids combine high thermal and electrochemical stability but suffer from high viscosity and low conductivity.^82^ Despite their advantages, liquid electrolytes still face several challenges such as leakage, flammability, narrow electrochemical windows, and instability when in contact with lithium metal.^83^ In this context, MXenes are expected to improve both the performance and stability of batteries when they are added to a liquid electrolyte system owing to their strong interaction with metal ions and their ability to guide ion movement.^84,85^ Specifically, the surface functional groups of MXenes, such as –O and –OH, show a strong affinity for metal ions, thus helping to uniformly guide ion flow and promote the formation and deposition of metals, which is beneficial for reducing uncontrolled dendrite formation. With regard to safety and flammability, MXenes act as both a matrix reinforcer and passive fire-retardant agent. Conventional organic carbonate electrolytes are inherently flammable and pose significant risks of thermal runaway.^86,87^ Therefore, MXenes have been incorporated into inherently safer solvent systems, such as ionic liquids (ILs) and aqueous electrolytes, which dramatically reduce volatility and fire hazards while maintaining ionic transport.^88,89^ In hybrid systems, the metallic nature and relatively high in-plane thermal conductivity of MXenes promote more efficient heat dissipation, helping mitigate localized hotspots.^90^ Crucially, under thermal abuse conditions, Ti_3_C_2_T_*x*_ can oxidize to TiO_2_, and this inorganic layer functions as a physical barrier that promotes char formation and contributes to self-extinguishing behavior in MXene-containing polymer matrices, thereby enhancing the overall battery safety.^89,91,92^ Additionally, MXenes directly address the narrow electrochemical stability window (ESW) limitation which restricts the achievable energy density. In aqueous electrolytes, the ESW is fundamentally constrained by the potential for water decomposition (∼1.23 V).^93^ When MXenes are combined with “water-in-salt” or other highly concentrated aqueous electrolytes, water molecules are strongly coordinated in cation solvation shells, lowering the effective water activity; together with the regulated interfacial reactions on MXenes, this kinetically suppresses parasitic reactions such as the hydrogen evolution reaction (HER).^44,94^ As a result, MXene-based aqueous micro-supercapacitors and asymmetric devices using water-in-salt-type electrolytes can operate stably at cell voltages up to ∼1.6–2.4 V, beyond the nominal 1.23 V limit.^44,94,95^ In non-aqueous, high-voltage applications, MXenes play a vital role in interface passivation as their abundant –F terminations promote the *in situ* formation of a mechanically robust and electronically insulating LiF-rich solid-electrolyte interphase (SEI) on Li or alloy-type anodes, homogenizing Li^+^ flux and suppressing continuous electrolyte decomposition.^90,96^ This stabilized interphase is essential for widening the oxidation limit, with advanced MXene-based hybrid polymer electrolytes demonstrating electrochemical stability windows up to ∼5.2 V *vs.* Li/Li^+^.^44,92,97^

The concentration of the dissolved conducting salt (*e.g.*, LiPF_6_, LiTFSI, and KFSI) is a fundamentally important variable that can be varied to balance kinetic performance, thermodynamic stability, and safety. Recent studies on advanced carbon electrodes for Li/K-ion- and Zn-based devices have consistently shown that changes in the salt concentration can markedly alter the rate capability, voltage efficiency, and cycling stability, indicating that the electrolyte concentration is a first-order design parameter rather than a secondary variable.^98–100^ In practice, lithium-based carbonate electrolytes are commonly formulated at concentrations of ∼1.0–1.2 M which offer high ionic conductivity and acceptable stability/cost, providing the optimal compromise between fast ion transport and manageable viscosity.^101,102^ Moreover, high-concentration electrolytes (HCEs) (≥3–5 M, and even >10 M in aqueous “water-in-salt” systems) restructure the primary solvation shell, yielding anion-rich local environments that broaden the electrochemical stability window and can enable otherwise unstable solvents (including water) by shifting decomposition pathways and stabilizing interphases.^101,103^ For example, ether HCEs such as 4–5 M LiFSI in DME form a robust, solvent-derived SEI and deliver stable Li cycling with high coulombic efficiency,^104,105^ even though increasing salt content rapidly increases viscosity and density, degrading wettability and rate performance, motivating the development of localized-high-concentration (LHCE) designs to maintain favorable transport.^101,104,105^ By contrast, low-concentration electrolytes (LCEs, typically <1.0 M and often ∼0.5–0.75 M) benefit from lower viscosity, better wettability, and faster ion-transport kinetics, and frequently yield a more organic-rich, elastic SEI that can better accommodate volume change (with system-specific trade-offs and need for additives).^106–108^ For example, studies of Na-ion batteries have reported effective operation in carbonate blends with concentrations in the ∼0.5–1.0 M range with additive-enabled interphase control, while optimized aqueous Zn systems commonly use ∼2–4 M ZnSO_4_ to balance transport with suppressed hydrogen evolution for long-life dendrite-free cycling.^109–111^ For potassium systems, very high-salt formulations such as 4 M KFSI in DME are now standard examples for stabilizing K metal and reducing polarization in organic media.^112,113^ In addition, LHCEs have been used to reduce viscosity and improve performance.^114^ MXene additives provide an approach for bypassing the kinetic tradeoff inherent in bulk concentration changes. Owing to the strong affinity of their –O and –OH surface terminations for cations, MXene nanosheets create a localized environment near the electrode surface that exhibits characteristics similar to those of an HCE. This environment promotes uniform ion flux and stable SEI formation, delivering the beneficial thermodynamic stabilization typically associated with bulk HCEs without imposing the bulk kinetic penalty of high viscosity.^44,47,115^ Experimental demonstrations in Zn and Li metal cells showed that MXene-containing electrolytes or separators induced uniform metal deposition, suppressed dendrites, and enabled long-term cycling, which is consistent with the localized HCE mechanism.^44,47,115,116^

**Fig. 5:**
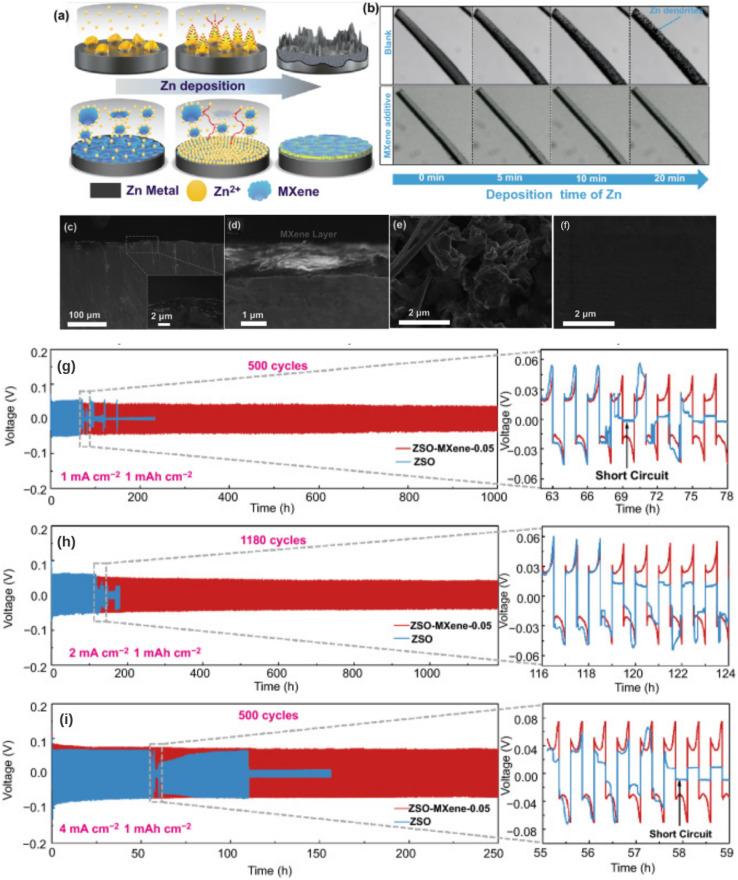
(a) Schematic of the Zn deposition process, comparing blank electrolyte (dendrite formation) with MXene-added electrolyte (uniform deposition); (b) time-lapse optical microscopy images showing Zn deposition over 20 minutes, visually demonstrating dendrite suppression with MXenes; (c–f) SEM images of Zn deposition layers and MXene layers; (g–i) voltage profiles of Zn–Zn symmetrical cells over multiple cycles (500, 1180, and 500 cycles) at different current densities (1, 2, and 4 mA cm^−2^).^47^ This figure has been reproduced from ref. 47 with permission from Springer Nature, copyright 2021.

Among MXenes, Ti_3_C_2_T_*x*_ has shown remarkable performance as an additive in aqueous battery electrolytes, particularly for zinc metal batteries. When dispersed in an aqueous electrolyte, MXene nanosheets function as multifunctional stabilizers and mediators and are particularly effective in suppressing dendrite growth on metal anodes. In Zn-ion systems with ZnSO_4_ electrolytes, addition of a small concentration of Ti_3_C_2_T_*x*_ nanosheets (*e.g.*, a few tens of µg mL^−1^) can significantly improve zinc plating/stripping behavior. The –O groups on MXenes adsorb onto the Zn surface and effectively direct the Zn^2+^ ion flow, resulting in fine zinc deposition and a stable protective interface layer. In addition, their abundant zincophilic oxygen-containing groups induce uniform Zn^2+^ deposition, while the high electronic conductivity of MXenes promotes uniform current distribution. It is of equal importance that MXenes participate in the formation of a robust solid-electrolyte interphase on Zn. Research by Sun *et al.* demonstrated that the addition of Ti_3_C_2_T_*x*_ to 2 M ZnSO_4_ induced a stable inorganic-rich SEI on the Zn anode, suppressing dendritic growth and side reactions.^47^ As a result, Zn–Zn symmetric cells with MXene-containing electrolyte achieved a high coulombic efficiency of up to 99.7% and sustained dendrite-free cycling for over 1180 h (almost 1200 cycles) at 2 mA cm^−2^, far outperforming the baseline electrolyte (Fig. 5). The MXene additive effectively reduced the local Zn^2+^ concentration gradients at the interface and prevented the formation of loose “dead” zinc, enabling long-term reversible Zn plating/stripping. DFT calculations supported this finding, showing high energies of binding between Zn and various MXene terminations: –OH (−7.64 eV), –F (−18.35 eV), and –O (−26.33 eV). These findings provide a proof of concept that MXenes can act as an interfacial agent in aqueous electrolytes to stabilize metal anodes. This approach is attractive because it utilizes MXenes without requiring a separate membrane; however, it requires that MXenes remain well-dispersed and not irreversibly consumed in the SEI.

By contrast, extension of this strategy to organic electrolytes presents additional challenges owing to the hydrophilicity of MXenes and their potential restacking in low-polarity solvents. However, initial studies have indicated that MXene additives can also enhance the behavior of lithium metal anodes in conventional Li-ion battery electrolytes.^117^ Functional –F and –O terminations on MXenes are known to generate LiF-rich SEI components when in contact with Li metal. A LiF-dominated SEI is highly desirable for lithium metal batteries because LiF is electronically insulating, ionically conductive, and mechanically robust, which helps to homogenize Li^+^ flux and inhibit dendrites. Molybdenum-based MXenes (*e.g.*, Mo_2_Ti_2_C_3_T_*x*_) with higher fluorine content have been shown to produce a stable LiF-rich SEI (predominantly LiF/Li_2_CO_3_) on lithium, dramatically reducing the nucleation overpotential and extending cycling life.^96^ With this MXene interlayer, lithium metal cells achieved ∼544 cycles at 3 mA cm^−2^ with ∼99.8% coulombic efficiency, representing a dramatic improvement over the baseline Cu anode. In another study, MXenes were used as a substrate/current collector for Li, and the results suggest that Ti_3_C_2_T_*x*_ or F-terminated MXene particles that are well-dispersed in an organic electrolyte can decompose trace HF or interact with Li^+^ to enrich the SEI in LiF. Additionally, a thin stoichiometric Ti_3_C_2_T_*x*_ (S-Ti_3_C_2_T_*x*_) coating on Cu supplies lithiophilic sites and –F/–O terminations that participate in LiF-rich SEI formation, lowering the nucleation overpotential and yielding uniform plating/stripping in anode-free formats, achieving an average coulombic efficiency of 98.2% after 100 cycles.^118^ Beyond interlayers, composite architectures such as MXene/g-C_3_N_4_ or MXene/COF build uniform artificial SEI layers that shield Li from corrosion and guide homogeneous Li deposition, boosting coulombic efficiency to ∼98.4% and sustaining >400 cycles.^53,119^ Three-dimensional supports, such as MXene-BN/Cu or Zn@MXene-coated Cu, further mitigate volume changes, enhance mechanical robustness, and maintain long-term stable operation (*e.g.*, ∼98% CE over 500 cycles for MXene-BN/Cu).^120,121^ Similar approaches have been employed for sodium and potassium metal batteries. MXene/CNT “nano-accordion” frameworks act as sodiophilic hosts that accommodate Na, distribute current, and restrain dendrite growth at high rates, enabling durable Na plating/stripping in carbonate/ether media.^122^ For K metal, 3D alkalized Ti_3_C_2_ nanoribbon frameworks provide abundant K-nucleation sites and mechanical confinement, yielding dendrite-free K deposition and improved cycling stability in organic electrolytes.^123^ Moreover, MXene sheets can also modulate the ion flow near the electrode; for example, Tian *et al.*^124^ reported a flexible MXene@Zn interlayer that not only functioned in aqueous systems, but also improved Li metal cycling in standard carbonate electrolytes by acting as an ionic redistributor and protecting layer. Overall, direct MXene addition to carbonate solvents (*e.g.*, EC/DMC) or ether-based electrolytes (DOL/DME) remains less explored than the MXene addition to aqueous solvents. Current studies are limited and no systematic data are available regarding long-term stability, apparently due to the difficulties involved in the dispersion of MXene sheets and concerns regarding their reactivity. To address this issue, researchers have developed surface functionalization, intercalation, solvent optimization, composite/doping, and oxidation-control routes for dispersing MXenes in organic electrolytes. One approach is to graft organic groups or polymer chains onto Ti_3_C_2_T_*x*_ (*e.g.*, aryl-diazonium, silane; PMA/PMMA grafts) to increase organophilicity and suppress restacking.^125–127^ For example, Usman *et al.* (2024) used an aryl-diazonium initiated acrylic-acid graft to form organophilic MXenes with stable dispersions in DMF/NMP and reduced re-aggregation, while Zhang *et al.* (2020) demonstrated that diazonium–amidoxime grafting improves solvent compatibility and colloidal stability of Ti_3_C_2_T_*x*_.^128,129^ Another strategy involves the use of ion exchange or intercalation to render MXenes more organophilic: exchanging MXene's interlayer cations with bulky organic cations or intercalating DMSO can enlarge the interlayer spacing, making it harder for sheets to restack when they are transferred to organic media.^130–132^ Fan *et al.* (2021) demonstrated divalent-cation-assisted MXene gelation, in which MXenes were assembled into a 3D network that resisted restacking.^133^ These preformed MXene frameworks can be infused with a liquid electrolyte to effectively create a percolating MXene scaffold within the cell. Additionally, ultrasonication and cosolvent techniques have been employed; dispersing MXenes in a polar aprotic solvent, such as NMP or DMF (in which MXenes show moderate solubility), and then mixing with the battery electrolyte can yield a stable suspension.^134^ Low concentrations (a few wt%) of MXenes are typically sufficient; at these levels, the risk of electronic short-circuiting is minimal, and MXene flakes remain separated by solvent molecules.^135,136^ In summary, although dispersion in organic electrolytes is nontrivial, practical strategies such as surface grafting, ion intercalation, and the use of polar cosolvents have been successful in mitigating MXene restacking. These measures ensure that the large surface area and functionality of MXenes are retained in the electrolyte, thereby fully realizing their benefits for liquid-cell applications.

**Fig. 6:**
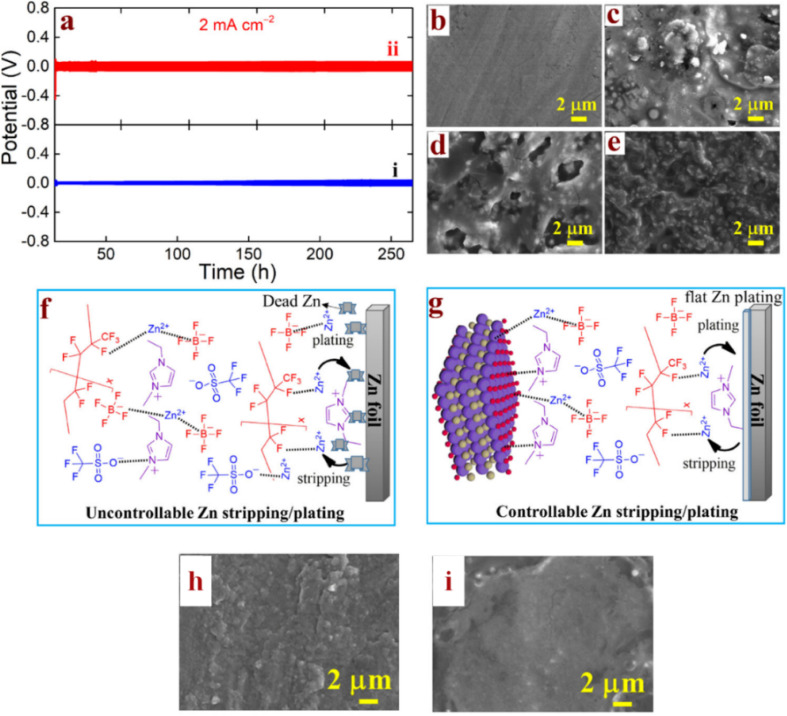
(a) Long-term Zn plating/stripping voltage profiles at 2 mA cm^−2^: (i) EMIMBF_4_/Zn(OTF)_2_/PVDF-HFP and (ii) Ti_3_C_2_T_*x*_/EMIMBF_4_/Zn(OTF)_2_/PVDF-HFP. (b) SEM image of pristine Zn foil; (c) Zn foil after cycling with EMIMBF_4_ GPE; (d) and (e) Zn foil after cycling with Ti_3_C_2_T_*x*_-containing GPE. (f) Schematic illustration of uncontrolled Zn stripping/plating in IL-only GPE. (g) Schematic of stabilized Zn deposition with Ti_3_C_2_T_*x*_-assisted interfacial regulation. (h) Post-cycling GPE surface (without MXene); (i) post-cycling GPE surface (with Ti_3_C_2_T_*x*_).^138^ This figure has been reproduced from ref. 138 with permission from American Chemical Society, copyright 2024.

Ionic liquids (ILs) provide a stable and compatible platform for MXene integration. One approach is to pre-intercalate IL cations into MXene layers, creating an MXene-IL hybrid that exhibits improved ionic transport (“ambipolar” electrode behavior in the supercapacitor context).^137^ In battery-oriented research, a semi-solid electrolyte was developed where Ti_3_C_2_T_*x*_ MXene was entrapped in a PVDF-HFP polymer gel containing a Zn^2+^-based ionic liquid (EMIMBF_4_ with Zn(OTF)_2_).^138^ The molecular-level mixing of MXenes, IL, salt, and polymer yielded the GPE homogeneous composite (discussed further in the next section) that effectively controlled Zn^2+^ transport. In zinc symmetric cells, the MXene/IL-containing electrolyte exhibited excellent Zn^2+^ mobility toward both the anode and cathode without the parasitic reactions (*e.g.*, corrosion and hydrogen evolution) that normally plague aqueous acidic Zn electrolytes. The results of post-mortem analysis shown in Fig. 6 confirm the presence of minimal byproducts and smooth Zn deposits after cycling with the MXene-IL gel electrolyte. This implies that MXenes can help mediate interfacial chemistry, even in IL-based systems, possibly through the interactions of their surface functional groups with ionic liquid components and metal ions. Generally, IL-grafted MXenes are an emerging concept; by covalently attaching ionic liquid chains onto MXene surfaces, researchers aim to create hybrid electrolytes that combine the ionic conductivity of ILs with the mechanical reinforcement of MXene nanosheets. Such MXene-IL hybrids have been reported to achieve high Li^+^ transference numbers and stable cycling at high voltages (approaching the 5 V class) in solid–liquid hybrid cells. Thus, ionic liquid electrolytes represent a promising route for MXene integration, offering a synergistic approach to leverage the functionality of MXenes in a liquid-like environment while maintaining stability under extreme electrochemical conditions (*e.g.*, high voltage and wide temperature range).^138^

### 4.2. (Quasi)-solid-state electrolytes

#### 4.2.1 Polymer-based electrolytes

Polymer-based electrolytes are key materials for next-generation batteries owing to their higher safety, flexibility, and compatibility with high-energy-density electrodes compared with traditional liquid electrolytes. They exist in two main forms: gel polymer electrolytes (GPEs) and solid polymer electrolytes (SPEs).

Gel polymer electrolytes (GPEs) represent a crucial quasi-solid-state system that is fundamentally defined as a polymer matrix (*e.g.*, PVDF-HFP, PEO, PVA, methyl cellulose, and gelatin) swollen with a liquid electrolyte solution (*e.g.*, salt dissolved in water, organic carbonates (EC/DMC and FEC), or ionic liquids (EMIM-BF4)). This structure successfully combines the safety and mechanical stability of a solid framework with the high ionic conductivity of a liquid electrolyte (typically in the range of 10^−3^–10^−2^ S cm^−1^).^139^ The polymer matrix acts as a scaffold, providing mechanical strength, flexibility, and dimensional stability; it immobilizes the liquid phase, prevents leakage, and improves safety. Polymer chains form a three-dimensional network, which is often chemically or physically crosslinked and can be tuned for porosity and flexibility.^58^ The liquid electrolyte phase provides a medium for ion conduction, is absorbed or trapped within the polymer network, and typically consists of a salt (*e.g.*, LiPF_6_, NaPF_6_, ZnSO_4_, or Mg(Tf)_2_) dissolved in solvents such as water, organic carbonates (EC/DMC or FEC), or ionic liquids (*e.g.*, EMIM-BF_4_ or PYR_14_-TFSI). The degree of swelling and the uniformity of the liquid phase within the polymer matrix are critical for achieving high ionic conductivity and stable electrochemical performance.^139,140^ Structurally, some advanced GPEs feature bicontinuous architectures, in which both the polymer and gel phases form interconnected networks, increasing the number of ion-transport channels and shortening the diffusion paths.^140^ Ion transport mechanisms in GPEs include: (i) segmental motion, where ions hop along polymer chains, particularly in amorphous regions;^141^ (ii) solvent-mediated diffusion where in highly swollen gels, transport is dominated by the diffusion through the liquid phase and can be decoupled from the polymer segmental motion;^140^ and (iii) polymer–ion interactions, whereby functional groups on the polymer (*e.g.*, –OH and –COOH) coordinate with ions and modulate transference numbers and selectivity.^142^

The incorporation of Ti_3_C_2_T_*x*_ MXene into GPE is particularly beneficial for flexible and wearable batteries (zinc-ion batteries, zinc–air batteries, *etc.*) for which prevention of electrolyte evaporation and stabilization of the metal electrode are of paramount importance. As mentioned in the previous section, a composite GPE made of PVDF-HFP with embedded Ti_3_C_2_T_*x*_ and an ionic liquid (EMIMBF_4_) demonstrated excellent performance in Zn-ion batteries.^138^ Additionally, the mechanical reinforcement from MXenes achieving a measured tensile strength of ∼0.36 MPa and 23% elongation in the composite membrane enhanced the resistance to dendrite penetration.

**Fig. 7:**
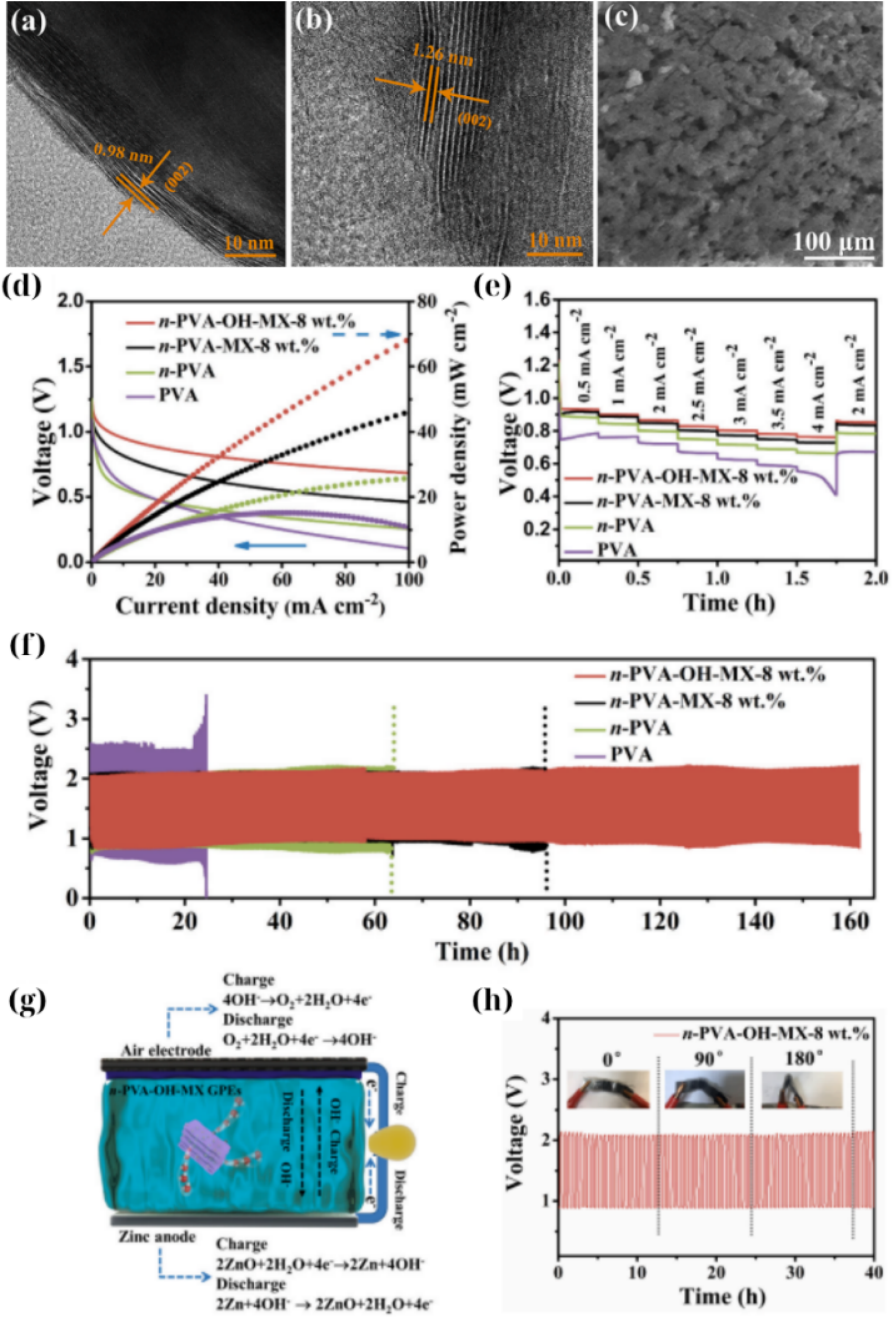
(a and b) HRTEM images showing the enlarged interlayer spacing of hydroxylated Ti_3_C_2_T_*x*_ MXenes (0.98 nm and 1.26 nm), indicating successful surface modification. (c) SEM image of the *n*-PVA-OH-MX-8 wt% membrane. (d) Discharge polarization curves and corresponding power density plots. (e) Rate performance of various GPEs at increasing current densities. (f) Long-term cycling stability of zinc–air batteries using different GPEs over 160 hours. (g) Schematic illustration of the electrochemical configuration and water-binding mechanism in the MXene-doped GPE. (h) Charge–discharge curves under different bending states (0°, 90°, and 180°).^143^ This figure has been reproduced from ref. 143 with permission from Elsevier, copyright 2022.

Based on these findings, Chen *et al.*^143^ presented an alkaline GPE including PVA with functionalized Ti_3_C_2_T_*x*_ that is useful for flexible Zn–air batteries. In this work, Ti_3_C_2_T_*x*_ was chemically hydroxylated (“alkalized”) to enrich its surface with –OH groups and then embedded in a poly(vinyl alcohol) gel containing KOH. The Ti_3_C_2_T_*x*_ formed a 3D porous network within PVA, which served as a water reservoir to greatly enhance water retention in the gel. Molecular dynamics simulations confirmed that the –OH-functionalized MXene had a stronger affinity for water molecules (contact angle ∼30°) than pristine MXenes (51°), explaining the improved hydration. As a result, the MXene-doped gel showed an ionic conductivity of 77.6 mS cm^−1^ that is significantly higher than that of the MXene-free gel and maintained its moisture over long-term operation. Zinc–air cells with MXene-GPE were cycled for over 160 h (at 2 mA cm^−2^; 15 min per cycle) prior to failure, roughly doubling the lifespan of the control cell without MXenes. Importantly, the strong PVA/MXene network kept the device mechanically flexible on bending by 90° showing little impact on the capacity (Fig. 7). Chen's work demonstrated that Ti_3_C_2_T_*x*_ can serve as a zincophilic scaffold (promoting uniform Zn plating) and a molecular sieve (binding water), thereby mitigating the primary drawbacks of alkaline GPEs, namely water evaporation and the formation of zinc dendrites. Subsequent studies further generalized this strategy to different MXene chemistries and electrolyte formulations for Zn-based and quasi-solid cells. Kumar *et al.*^138^ employed a Mo_2_CT_*x*_ MXene obtained *via* an electrochemical etching route and embedded it into an EMITFSI/Zn(OTF)_2_/PVDF-HFP gel polymer electrolyte, simultaneously achieving high ionic conductivity, higher Zn^2+^ transference number, and excellent Zn plating/stripping compatibility. The resulting CaV_6_O_16_·3H_2_O‖Mo_2_CT_*x*_-GPE‖Zn full cells delivered capacities of approximately 155 mAh g^−1^ with ∼99% capacity retention and near-100% coulombic efficiency over extended cycling, confirming that MXene-reinforced gels can sustain practical areal capacities in quasi-solid Zn metal batteries. Building on this approach, the same group recently reported a Ti_3_C_2_T_*x*_-filled EMIMBF_4_/Zn(OTF)_2_/PVDF-HFP semi-solid polymer electrolyte, where Ti_3_C_2_T_*x*_ sheets serve as both mechanical reinforcers and Zn^2+^ transport regulators.^138^ Zn‖Ti_3_C_2_T_*x*_/EMIMBF_4_-GPE‖Zn symmetric cells showed highly stable plating/stripping with low polarization, while Zn-based full cells maintained high reversibility and suppressed dendrite growth at practical current densities. These results clearly demonstrate that MXene-containing gel electrolytes can combine high Zn^2+^ conductivity, wide operating temperature windows, and long dendrite-free cycling, bringing Zn metal batteries closer to real-world conditions.

In addition to Zn systems, MXene incorporation has been shown to be beneficial for sodium-ion batteries. Wang *et al.* (2021)^144^ fabricated a Ti_3_C_2_T_*x*_-modified gel electrolyte for a Na metal battery (Na anode‖Na_3_V_2_(PO_4_)_3_ cathode). They dispersed Ti_3_C_2_T_*x*_ into a PVDF-HFP-based gel containing NaPF_6_ in organic solvent, improving the Na^+^ transference number and the mechanical strength. The conductive MXene network likely facilitated ion dissociation and reduced concentration polarization at the Na anode. The resulting Na battery showed a significantly enhanced rate capability and cycle life compared to a gel without Ti_3_C_2_T_*x*_ MXene. As a result, the Na metal battery demonstrated notably improved rate performance and long-term cycling stability, achieving 95% capacity retention over 300 cycles at 0.5C. However, more recent work has advanced MXene-based gel and quasi-solid electrolytes for Na systems much closer to practical application. For example, Chen *et al.* (2025)^145^ designed an MXene-enhanced PEGDA-crosslinked quasi-solid electrolyte for sodium-ion batteries, where a three-dimensional PEGDA/MXene/PVDF-HFP network immobilized the liquid phase while preserving high Na^+^ conductivity. With only ∼1.5 wt% Ti_3_C_2_T_*x*_, the quasi-solid electrolyte reached an ionic conductivity on the order of 10^−3^ S cm^−1^ at 30 °C and maintained a wide electrochemical stability window, enabling Na_3_V_2_(PO_4_)_3_‖Na full cells to deliver >100 mAh g^−1^ at multi-C rates and retain ∼90% of their capacity over thousands of cycles. This study also reported excellent flame-retardant behavior, with the MXene-reinforced membrane self-extinguishing rapidly after flame exposure, underscoring the dual role of MXenes as a mechanical/thermal stabilizer and ion transport promoter in advanced Na-based quasi-solid electrolytes.

A clear pattern has been demonstrated: Ti_3_C_2_T_*x*_ fillers in GPEs improve interfacial stability, reduce dendrites, and increase ionic conductivity. Mechanistically, multiple properties give rise to these beneficial effects.

(1) Reduced polymer crystallinity: MXene sheets hinder polymer chain packing and increase the number of amorphous regions for ion transport.^146^ In segmental-motion-dominated polymers, Li^+^, Na^+^ and Zn^2+^ hopping is strongly coupled to local chain dynamics; therefore, increasing the amorphous fraction not only increases the number of continuous ion-conducting pathways but also lowers the apparent activation energy for ion transport.^26,144,146,147^ When Ti_3_C_2_T_*x*_ is well-dispersed in polymer matrices such as PEO, PVDF-HFP or PVA, the 2D flakes intercalate between chains, disrupt long-range order and reduce crystallinity. This is often manifested in the results of the DSC/XRD measurements by suppressed melting peaks and broadened glass-transition features that are correlated with higher σ and improved rate capability.^26,147,148^ In other words, rather than MXenes acting only as an inert filler, their two-dimensional geometry and surface interactions actively reorganize the polymer microstructure into a more ionically conductive state.

(2) Improved mechanical modulus: even at low loadings, the high aspect ratio of MXenes leads to the formation of a reinforcing network. GPEs with MXenes exhibited higher tensile strength and puncture resistance which are crucial for resisting dendrite penetration in Zn or Na metal cells.^144,149–151^ From the perspective of mechanical behavior, dendrite growth requires metallic filaments to locally deform and fracture the electrolyte. By increasing the elastic modulus and fracture toughness due to presence of a percolated MXene skeleton, the GPE can sustain higher local stresses prior to cracking, effectively increasing the critical current density (CCD) for dendrite penetration.^149,152,153^ The 2D flakes also help maintain conformal contact with the metal surface during plating/stripping, reducing void formation and “hot spots” in the current distribution that would otherwise accelerate dendrite nucleation.^152,153^

(3) Space-charge layer effects: negatively charged MXene surfaces (particularly those with –O/–F terminations) tend to adsorb cations and repel anions.^144,154^ In GPEs, this can increase the effective cation transference number through local accumulation of cations on MXene surfaces.^88,144,155^ This mitigates the deleterious anion polarization at the metal anode, yielding a more uniform deposition.^144,155^ Based on the Poisson–Nernst–Planck model, MXene sheets generate space-charge regions in which the electrostatic potential and ion concentrations deviate from those of the bulk. These cation-rich “corridors” along the MXene sheets shorten the diffusion length for charge compensation and reduce the concentration gradients near the electrode under applied current.^120,144,149^ As a result, cells with MXene-doped GPEs exhibit smaller concentration overpotentials, more stable voltage plateaus, and delayed onset of cell failure at high current densities, consistent with a genuine increase in effective *t*^+^ (*e.g. t*^+^ = 0.558 for Na^+^; *t*^+^ = 0.76 for Li^+^) and suppression of anion crowding at the interface.^144,149,153^

(4) SEI/CEI formation: MXenes participate in interphase chemistry. For instance, Ti_3_C_2_T_*x*_ contains oxide terminations that may form M–O–M networks with the polymer or react with the electrolyte components to produce a stable SEI rich in inorganic compounds (*e.g.*, LiF, Li_2_CO_3_, ZnO, and NaF).^156,157^ In Li-based systems, the –F terminations act as an internal fluorine source; their reduction in contact with Li promotes LiF-SEI domains that are electronically insulating but Li^+^-conductive and mechanically stiff. This combination decouples ion transport from electron leakage, homogenizes Li^+^ flux, and mechanically “armors” the interface against tip growth of dendrites.^149,153^ In Zn or Na cells, Ti_3_C_2_T_*x*_ MXene-derived oxides (*e.g.*, TiO_*x*_) and hydroxides can similarly be integrated into an inorganic-rich interphase that suppresses parasitic reactions (corrosion and hydrogen evolution) and stabilizes metal stripping/plating for hundreds of hours.^47,158^ Chen *et al.*^143^ observed that the addition of MXenes to a Zn–air GPE led to a smoother and more compact Zn|electrolyte interface after cycling, indicating a modified SEI. The effect of MXenes can be compared to that of alternative fillers such as the widely used graphene oxide (GO) 2D additive which has been reported to improve both ionic conductivity and mechanical strength in polymer electrolytes by interacting with the host polymer and creating ion-transport pathways.^159,160^ MXenes are intrinsically metallic and exhibit high electrical conductivities owing to their strong M–X bonds, which allow them to function as highly efficient electron-conductive bridges. These bridges are vital for homogenizing the current density distribution across the electrode surface, which is essential for preventing localized high current densities that lead to heterogeneous plating and eventual metal dendrite nucleation.^161^ By contrast, GO is electrically insulating, so unlike MXenes, it cannot provide such electron-conductive “bridges” that promote a homogeneous current density.^162^ Furthermore, the surface chemistry of MXenes, particularly their abundant fluorine terminations, directly facilitates *in situ* formation of a compact, durable, and LiF-rich SEI layer. This LiF-rich SEI is highly desirable because it suppresses parasitic reactions between the electrode and electrolyte, enhances interfacial stability, promotes uniform lithium deposition, and ultimately improves the cycling stability and coulombic efficiency.^96,156,163^ By contrast, GO lacks reactive –F terminations and therefore does not promote the formation of a LiF-rich SEI on Li metal anodes.^157^ Instead, GO and reduced GO can only support the LiF-rich SEI indirectly, and through careful engineering of their size and defect density mainly act as high-surface-area scaffolds that facilitate Li-ion transport and provide additional interfaces for SEI growth, rather than serving as an active fluorine source.^164–166^ As a result, the LiF-rich SEI formed in the presence of MXenes is typically more robust and uniform because rather than relying solely on salt or solvent decomposition as the fluorine source for the SEI, the fluorine from the MXene surface actively and directly contributes to the SEI composition.^165,166^ This fundamental difference ensures that MXenes simultaneously shape the ionic landscape (through space charge and SEI chemistry) and the electronic landscape (through its metallic conductivity), enabling coupled electro-ionic regulation that is impossible to achieve using GO-based systems. Of course, some tradeoffs are involved in the use of MXenes which are not silver bullets.

To date, most reports of highly impressive electrolyte performance still rely on Ti_3_C_2_T_*x*_ produced by HF or *in situ* HF (LiF/HCl) etching, which is intrinsically hazardous, costly (lab-scale estimated cost is on the order of a few hundred US dollars per kilogram of dry MXene), and generates fluorinated waste streams that are difficult to manage at scale.^40,167^ In addition, Ti_3_C_2_T_*x*_ is highly sensitive to moisture and oxygen, so that progressive oxidation to TiO_2_ during long-term processing or storage gradually erodes electrical conductivity and alters surface terminations, precisely undermining the interfacial and transport functions that MXenes are meant to provide.^168^ While fluoride-free synthesis routes, such as electrochemical, Lewis-acid molten-salt, and alkali-hydrothermal etching, are emerging, their delamination quality, termination control, and batch-to-batch reproducibility still lag behind those of the HF benchmark, representing a major bottleneck for realistic solid-electrolyte manufacturing.^169^

The same mechanism can be applied to solid polymer electrolytes (SPEs), which are solvent-free polymer matrices. SPEs offer higher safety and enable thinner electrolyte layers for greater energy density; however, they often suffer from low room-temperature conductivity and poor electrode contact. The integration of Ti_3_C_2_T_*x*_ MXene into SPEs has emerged as a powerful strategy to address these issues. MXene fillers can improve ionic conductivity by disrupting polymer crystallinity and providing conduction pathways, and can reinforce the polymer to suppress dendrite penetration and improve interfacial contact.^26^ Moreover, advanced surface engineering of MXenes (such as polymer grafting) enables strong compatibility with polymer hosts, leading to a uniform filler distribution even at relatively high loadings.

**Fig. 8:**
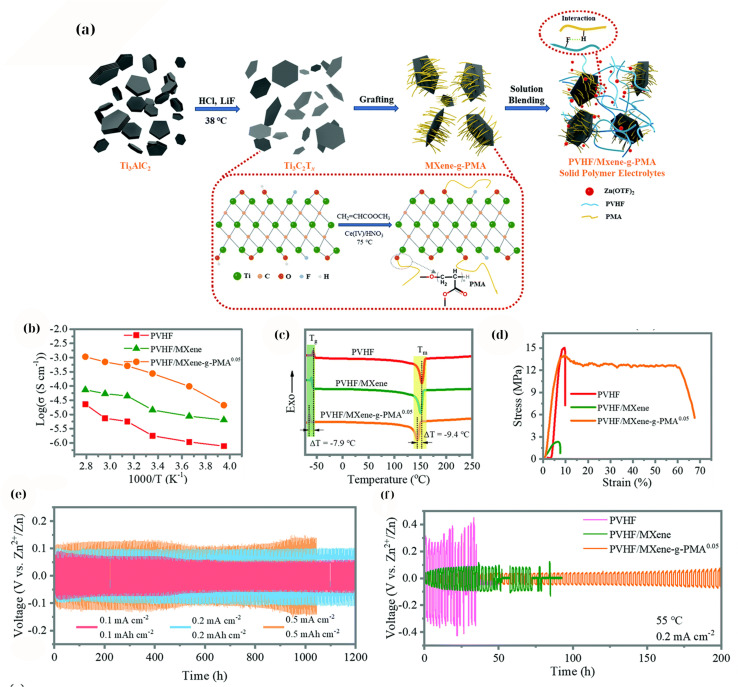
(a) Schematic of PMA-grafted Ti_3_C_2_T_*x*_ and PVHF/MXene-*g*-PMA SPE; (b) temperature-dependent ionic conductivity (Arrhenius) showing enhanced *σ* for grafted MXenes; (c) DSC traces evidencing reduced crystallinity; (d) stress–strain curves indicating improved mechanical robustness; (e) long-term Zn∥Zn cycling at room temperature; (f) stable cycling at 55 °C.^170^ This figure has been reproduced from ref. 170 with permission from The Royal Society of Chemistry, copyright 2021.

A seminal study conducted by Chen *et al.*^170^ demonstrated a highly effective SPE for solid-state Zn batteries using a PVDF-HFP polymer filled with poly(methyl acrylate)-grafted Ti_3_C_2_T_*x*_ MXene. By grafting PMA brushes onto MXene surfaces, Chen *et al.*^170^ achieved excellent MXene dispersion within the PVDF matrix owing to the favorable interactions between PMA and PVDF-HFP. The resulting composite (denoted as PVHF/MXene-*g*-PMA) exhibited an ionic conductivity of 2.69 × 10^−4^ S cm^−1^ at room temperature, which is three orders of magnitude higher than that of the plain PVDF-HFP polymer without the MXene filler. This extraordinary enhancement can be attributed to the following effects: (i) the well-dispersed 2D MXene sheets significantly suppress polymer crystallization and create continuous ion conduction channels, and (ii) the grafted PMA likely immobilizes anions or creates percolating ion-conductive pathways at the MXene-polymer interface. In Zn–Zn symmetric cell tests, the SPE enabled dendrite-free Zn plating/stripping for over 1000 h at room temperature, and even at 60 °C the cell ran stably for ∼200 h without short-circuiting (Fig. 8). The SPE also effectively eliminated side reactions such as hydrogen evolution and Zn passivation, leading to a shelf life of >90 d for the solid-state Zn cell (*i.e.*, the cell can be stored at −35 °C to 100 °C for three months and still operate). Furthermore, solid Zn-ion full cells (using a MnO_2_-based cathode) with this MXene-PMA/PVDF SPE achieved 10 000 cycles at 2C with minimal capacity fading. These results indicate that properly engineered MXene fillers can address two of the greatest challenges in SPEs, namely low ionic conductivity and poor Zn anode stability. The MXene-*g*-PMA filler ensured a high Zn^2+^ transference number and smooth Zn^2+^ plating at the interface (likely due to a favorable MXene-induced SEI and a more uniform electric field distribution). Notably, this SPE allowed the Zn anode to be cycled at high utilization (thin 20 µm Zn foil was used, representing ∼30% utilization, much higher than the typical utilization reported in solid-state studies) without shorting. The robust MXene network prevented dendrite penetration and obviated the need for external pressure or interface wetting layers, thereby simplifying the cell assembly.

**Fig. 9:**
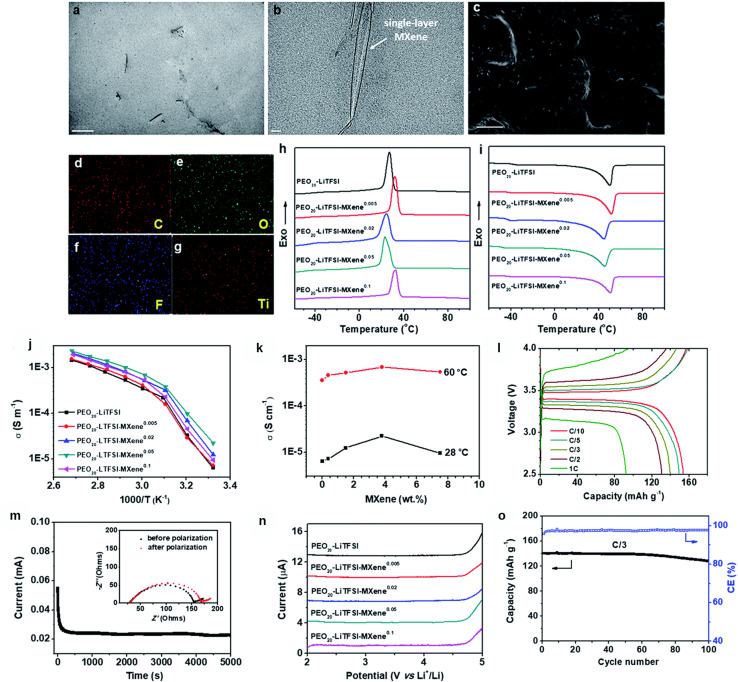
(a) Low-magnification TEM image of Ti_3_C_2_T_*x*_ flakes dispersed in the polymer; (b) HRTEM image of a single-layer MXene; (c) SEM morphology; (d–g) EDS maps of C, O, F, and Ti confirming the composition and distribution; (h and i) DSC thermograms showing suppressed PEO crystallinity and shifted transitions upon MXene addition; (j) Arrhenius plots of ionic conductivity; (k) ionic conductivity *vs.* MXene loading at 28 °C and 60 °C showing a low-wt% optimum; (l) galvanostatic profiles of LFP∥Li at various C-rates; (m) Bruce–Vincent polarization for *t*^+^ with the Nyquist inset; (n) linear sweep voltammetry of oxidative stability; (o) cycling performance of LFP∥Li at C/3 with coulombic efficiency.^26^ This figure has been reproduced from ref. 26 with permission from The Royal Society of Chemistry, copyright 2019.

MXenes have also been investigated as classic PEO (polyethylene oxide)-based solid electrolytes for lithium metal batteries. Pan *et al.*^171^ reported a study where low loadings of Ti_3_C_2_T_*x*_ MXene (∼1–4 wt%) were uniformly blended into PEO-LiTFSI to create a MXene-containing polymer electrolyte (denoted as MCPE). The introduction of 2D MXenes had a pronounced effect; it simultaneously retarded PEO crystallization and enhanced polymer segmental motion, both of which are beneficial for Li^+^ transport. Compared to the addition of 0D (nanoparticle) or 1D (nanowire) fillers, 2D MXenes were markedly more effective in boosting ionic conductivity. With only 3.6 wt% MXene, the room temperature ionic conductivity reached 2.2 × 10^−5^ S cm^−1^ (at 28 °C), a respectable value for a PEO-based SPE. Even the addition of 1.5 wt% MXene was sufficient to improve the cycling performance of Li metal cells on par with that of the state-of-the-art composite electrolytes that often use ceramic fillers or ionic liquids. The Li‖Li symmetric cells and Li‖LiFePO_4_ cells using the MXene/PEO SPE showed stable cycling which was attributed to the MXene's ability to both accelerate Li^+^ conduction and form a stable interface with lithium. Essentially, MXene's 2D geometry and functional surfaces provide what one may call “ion highways” through the polymer and also likely generate a Li-friendly SEI (rich in Li salt decomposition products anchored to MXenes). Additionally, the high aspect ratio of MXenes means that they can form a connected network at low volume fractions which can also improve the mechanical stiffness of the polymer and hence resist dendrite growth. This provides a distinct advantage over the traditional ceramic fillers which often require much higher loadings (20–30%) to form percolation networks (Fig. 9).

**Fig. 10:**
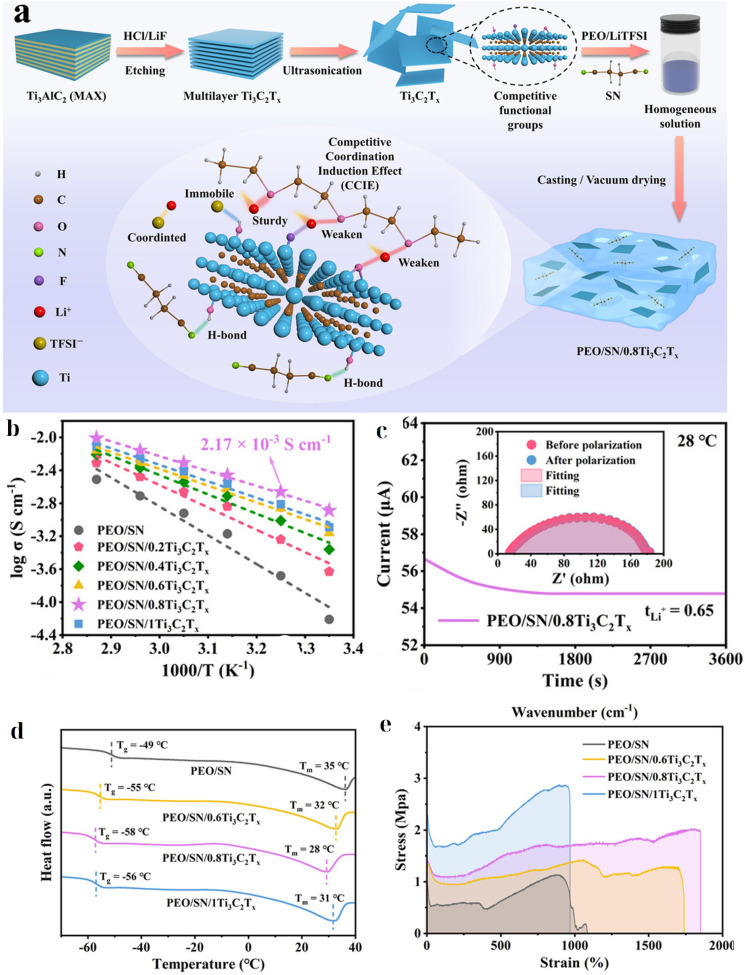
(a) Schematic of the PEO-SN/MXene co-matrix and CCIE; (b) Arrhenius conductivity highlighting ∼2.17 × 10^−3^ S cm^−1^ at 35 °C; (c) Li^+^ transference (Bruce–Vincent); (d) DSC evidence of reduced crystallinity; (e) stress–strain curves showing improved mechanical robustness.^172^ This figure has been reproduced from ref. 172 with permission from Elsevier, copyright 2025.

Recent studies have reported improvements in the conductivity and stability of MXene-enhanced SPEs. By introducing a succinonitrile (SN) plastic-crystal co-matrix into the PEO host alongside Ti_3_C_2_T_*x*_, Xu *et al.*^172^ reported a PEO-SN-based SPE that achieved Li^+^ conductivity on the order of 10^−3^ S cm^−1^. Specifically, at 35 °C their MXene-enhanced polymer showed a conductivity of ∼2.17 × 10^−3^ S cm^−1^, a remarkably high value for a polymer electrolyte at near-ambient temperature. The high conductivity was accompanied by stable interfacial resistance against the lithium metal anode owing to the ability of MXenes to form a robust interphase. The incorporation of succinonitrile (a plastic crystal) along with MXenes likely helped achieve such high conductivity by further reducing the crystallinity and providing faster segmental dynamics. Mechanical properties and Li interface stability were improved by the addition of MXenes. Li symmetric cells with the MXene-PEO electrolyte showed long-term cycling without short-circuiting, indicating suppression of dendrite growth (Fig. 10).

Beyond serving as a passive filler or interfacial regulator, MXenes can also be chemically engineered as a carrier for ionic liquids (ILs) to create ion-selective, high-voltage-stable polymer electrolytes; by tethering IL moieties directly onto Ti_3_C_2_T_*x*_, the MXene surface localizes mobile species at 2D interfaces, immobilizes anions, and improves compatibility/dispersion in polyethers, thereby decoupling ionic conductivity gains from the plasticization penalties observed in bulk IL blends. Qian *et al.* (2025)^97^ grafted an IL onto Ti_3_C_2_T_*x*_ and dispersed this IL-functionalized MXene (MXene-IL) in a PEO matrix, achieving high ionic conductivity (∼7.2 × 10^−4^ S cm^−1^ at 60 °C) and a notably high Li^+^ transference number (∼0.51). By anchoring TFSI^−^ on the IL chains attached to MXenes, the composite promoted selective cation transport, while the electrochemical stability window widened to ∼5.2 V, indicating that the IL grafts helped passivate interfaces and suppress high-voltage degradation. In LiFePO_4_‖Li full cells, the MXene-IL SPE sustained ∼155 mAh g^−1^ over 120 cycles with >95% retention, reinforcing the broader design principle that surface-tailored MXenes (through IL grafting in this case) can combine the advantages of polymers, MXenes, and IL to enhance SPE performance.

Overall, MXenes deliver outsized gains even at low loadings (a few wt%), with multiple studies showing optimal performance in the low single-digit range (∼1–5 wt%). However, two practical challenges remain to be addressed.

First, stability: Ti_3_C_2_T_*x*_ is prone to oxidation to TiO_2_, particularly in water or under high potentials, which degrades electrical conductivity and surface functionality; while partial controlled oxidation can sometimes be beneficial (*e.g.*, through forming a stiff TiO_2_ scaffold in Zn systems^38^), uncontrolled oxidation erodes performance over time. Encapsulation and oxygen-free processing (*e.g.*, using antioxidants such as sodium l-ascorbate) and high-permittivity solvents have been shown to mitigate degradation.^149,173^

Second, dispersion: MXene nanosheets tend to restack *via* van der Waals forces, particularly in low-polarity media, leading to aggregates that decrease the benefits provided by MXenes. Because MXenes are highly conductive, they can create electronic pathways inside solid electrolytes.^174,175^ Thus, the filler fraction must be kept near the optimum: MXene networks exhibit ultralow electrical percolation thresholds in polymers (∼0.03–0.05 vol%), so that excessive loading risks electronic percolation and internal shorting; for SPEs, the membrane must remain electronically insulating (very low *σ*_e_) to avoid dendrite growth.^176^ However, from a practical standpoint, the “conductivity paradox” of MXene-filled electrolytes deserves more attention. Most studies deliberately maintain the MXene loading below the electronic percolation threshold (typically <5 wt%) to prevent the formation of a continuous electron-conducting network across the electrolyte. Although this strategy is effective in suppressing short circuits and self-discharge, it also limits the extent of mechanical reinforcement and interfacial engineering that can be achieved. In our view, a promising direction is to move toward “electronically insulated MXenes,” for example by conformally coating Ti_3_C_2_T_*x*_ with insulating polymers or by intentionally oxidizing the outermost shell into TiO_2_-like domains. Such core–shell or gradient structures would, in principle, allow higher filler loadings to be used to toughen the membrane and modulate the interfaces without introducing an unwanted electronic leakage pathway.

#### 4.2.2 Inorganic and composite electrolytes

Traditional polymer–ceramic composite electrolytes (*e.g.*, PEO–LLZO) are limited by the rigid, dissimilar surfaces of ceramic fillers such as LLZTO which despite high bulk Li^+^ conductivity (∼10^−3^ S cm^−1^) result in poor interfacial contact and high interfacial resistance (hundreds–thousands of Ω cm^2^).^177,178^ MXenes address this critical issue by functioning as a compliant, multifunctional interfacial bridge that fundamentally improves contact and transport through a synergistic mechanism. These dual synergistic roles are described as follows. First, interfacial contact is enhanced. The most important function of MXenes is to act as a flexible lamellar coupling agent. Unlike rigid 0D ceramic nanoparticles, the 2D MXene nanosheets conform and “wet” the irregular surfaces of both the rigid ceramic fillers and the electrode materials. Such wetting effectively bridges the microscopic gaps inherent in solid–solid interfaces, drastically reducing the intrinsic high interfacial resistance to desirable values (typically below 100 Ω cm^2^) and ensuring intimate continuous contact throughout mechanical cycling.^26,179^ For instance, incorporation of MXenes at the Li/LLZO interface has been shown to reduce resistance from ∼1291 Ω cm^2^ to as low as 5–33 Ω cm^2^.^179^ Furthermore, the surface functional groups of MXenes (–OH, –F, –O) facilitate strong non-covalent interactions (such as H-bonds) with both the polymer host and the ceramic surface, further enhancing the stability and lowering the impedance.^174^ Second, the dual conduction pathways are optimized. The composite structure forms a percolated network that optimizes both ionic and electronic transport. While the ceramic phase provides a fast, high-conductivity pathway for bulk Li^+^ transport, metallic MXenes simultaneously create electron-conductive networks that support uniform current distribution and suppress dendrite formation.^26,152,179^ Crucially, the negatively charged MXene surface promotes efficient Li^+^ transfer across the interface, acting as local cation-rich pathways and significantly enhancing the overall effective cation transference number *t*^+^. This synergistic network yielded composites with exceptionally high performance, including an ionic conductivity as high as 14.8 mS cm^−1^ and *t*^+^ as high as 0.91.^152,179^

**Fig. 11:**
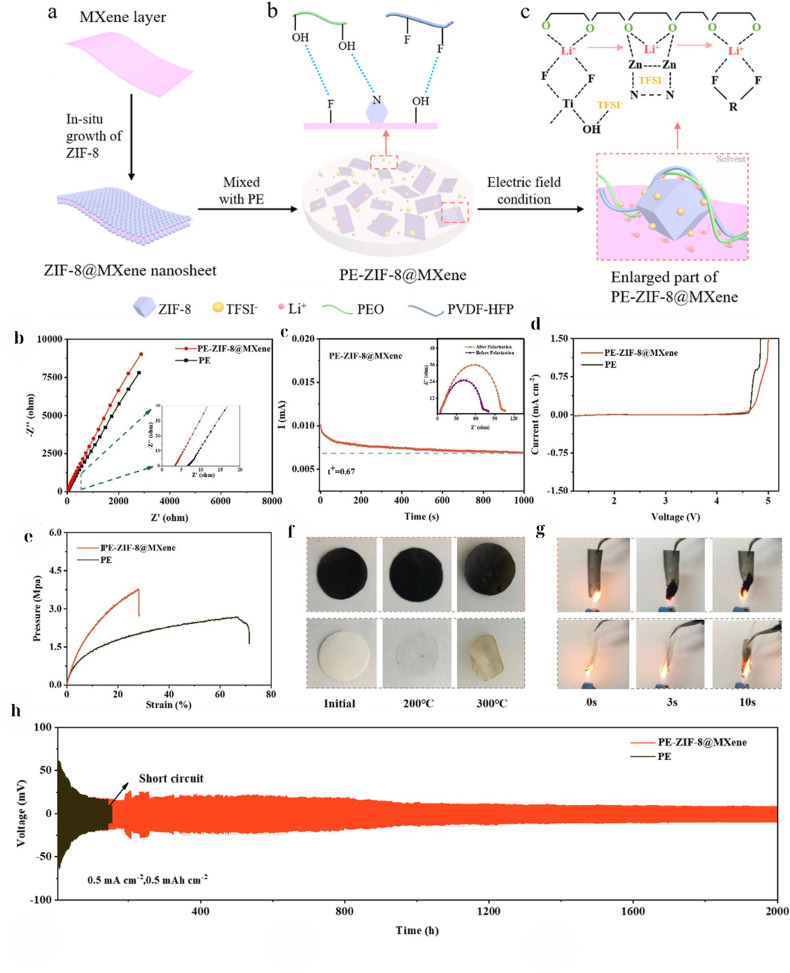
(a) *In situ* growth of ZIF-8 on MXenes; (b) mixing with PE under an electric-field alignment; (c) enlarged view of interfacial interactions (H-bonding/solvation); (d) impedance-derived ionic conductivity; (e) Bruce–Vincent polarization showing Li^+^ transference; (f) tensile stress–strain; (g) thermal/flame tests; (h) long-term Li∥Li symmetric cycling (∼2000 h at 0.5 mA cm^−2^).^153^ This figure has been reproduced from ref. 153 with permission from Elsevier, copyright 2022.

This architectural synergy was demonstrated in several leading systems. For instance, Zhao *et al.*^153^ anchored ZIF-8 nanoparticles on Ti_3_C_2_T_*x*_ and dispersed this hybrid in a PEO matrix. The MXene provided electronic conductivity and mechanical reinforcement, while the MOF particles offered fast Li^+^ transport pathways and absorbed thermal energy (improving safety). As shown in Fig. 11, the result was a “tri-component” electrolyte (PEO-MXene-MOF) that exhibited performance superior to those of both PEO-MXene and PEO-MOF binary composites. In this system, MXenes provide electrical conductivity and mechanical reinforcement, whereas MOF particles create fast Li^+^ transport channels and absorb thermal energy, thereby enhancing safety. As a result, the PE-ZIF-8@MXene electrolyte exhibits a high ionic conductivity (4.4 mS cm^−1^), a large Li^+^ transference number (0.76), improved tensile strength, and outstanding flame-retardant capability. The Li‖PE-ZIF-8@MXene‖Li symmetric cell operated stably for up to 2000 h, and the Li‖PE-ZIF-8@MXene‖LiFePO_4_ full cell retained 89.6% of its capacity after 500 cycles, outperforming the binary systems containing only MXenes or MOFs. Recently, Cheng *et al.*^180^ reported ultrathin PVDF-HFP CPEs where ZIF-8 or UiO-66 particles are pre-impregnated with LiTFSI so that the MOF nanochannels act as “ionic highways,” boosting salt dissociation and yielding *σ* ∼ (2.3–3.4) × 10^−4^ S cm^−1^ at RT, *t*^+^ as high as 0.90, and an ESW ∼4.9 V; it was also found that ∼14 µm films show self-extinguishing flame behavior and a tensile strength of ∼9 MPa with high strain. Symmetric Li∥Li cells can be cycled for >1500 h with low overpotential, LiFePO_4_ cells retain 94.6% capacity after 300 cycles (1C), and an Ah-level pouch retains a capacity of 702 mAh after 200 cycles, demonstrating that MOF channels and the polymer deliver uniform Li^+^ flux and stable interfaces. This directly supports the tri-component design (MOF provides fast Li^+^ transport pathways and safety, and MXenes supply electronic/thermal spreading and mechanical reinforcement) and suggests that the use of pre-salt-loaded MOFs combined with MXene co-fillers is a promising route for coupling ionic highways with current homogenization in next-generation solid electrolytes. However, such “headline” improvements are highly sensitive to the test conditions. For fair cross-study comparison, conductivity should be normalized by temperature (preferably reported at 25 °C with Arrhenius/VTF analysis), film thickness and density should be stated explicitly, and the cell geometry (blocking *vs.* Li∥Li; symmetric *vs.* full cell) and stack pressure should be carefully controlled; otherwise, plasticization or thickness effects can masquerade as intrinsic conductivity gains. Similarly, many MXene-based electrolytes have been claimed to “suppress dendrites” on the basis of relatively mild symmetric-cell tests (<0.5 mA cm^−2^, <100 h, thick Li foil and excess electrolyte). Future work needs to systematically probe higher current densities (>5 mA cm^−2^), high depth of discharge, lean-electrolyte conditions and anode-limited designs to establish whether MXene-enabled architectures truly deliver dendrite-free cycling under practical conditions.

**Fig. 12:**
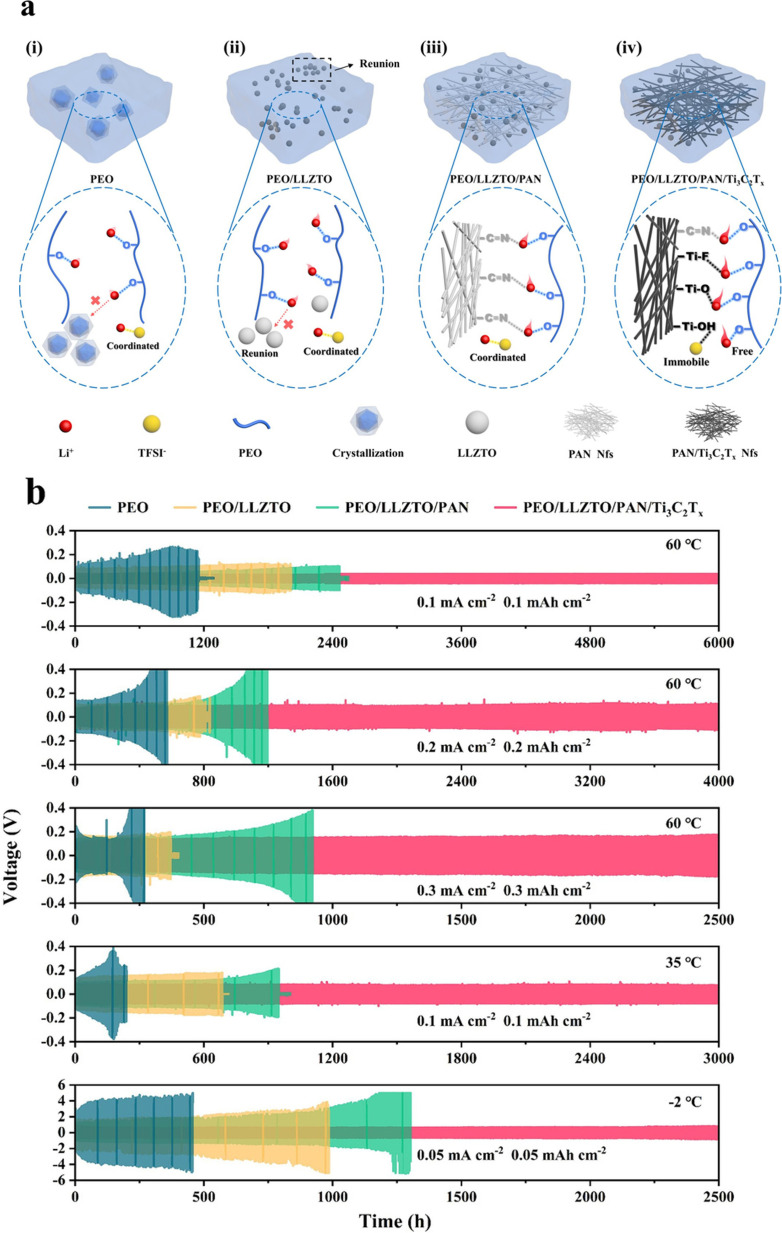
(a) Schematic Li^+^ transport and dendrite suppression; (b) Long-term Li∥Li cycling.^152^ This figure has been reproduced from ref. 152 with permission from Elsevier, copyright 2024.

Ceramic nanofillers such as LLZO (Li_7_La_3_Zr_2_O_12_) offer intrinsically high Li^+^ conductivity (∼10^−3^ S cm^−1^) and are widely used in polymer composites; yet they typically require high loadings and their rigid surfaces can hinder intimate interfacial contact.^181–183^ Here MXenes occupy a useful middle ground as their compliant lamellar sheets conform to electrode and filler surfaces and can act as “bridges” between dispersed ceramic particles, reducing interfacial resistance while simultaneously supporting ionic transport through the ceramic network, and improving interfacial contact and current distribution.^184^ For instance, Xu *et al.*^152^ combined electrospun MXene/PAN nanofibers with LLZTO nanoparticles in a PEO matrix. Here, the MXene/PAN network supplies a flexible, lithiophilic scaffold and additional interfacial transport sites, while LLZTO boosts the bulk Li^+^ conductivity. The composite achieves a tensile strength of ∼2.37 MPa and room-temperature ionic conductivity on the order of 10^−4^ S cm^−1^ (increasing at elevated temperature), translating to extended Li-metal cycle life and high critical current density (CCD). Mechanistically, the MXene/PAN fibers acted as a self-reinforcing and compliant framework that improved interfacial contact and guided uniform Li deposition, whereas the ceramic phase provided highways for fast ion transport, thus demonstrating effective division of labor (Fig. 12).

**Fig. 13:**
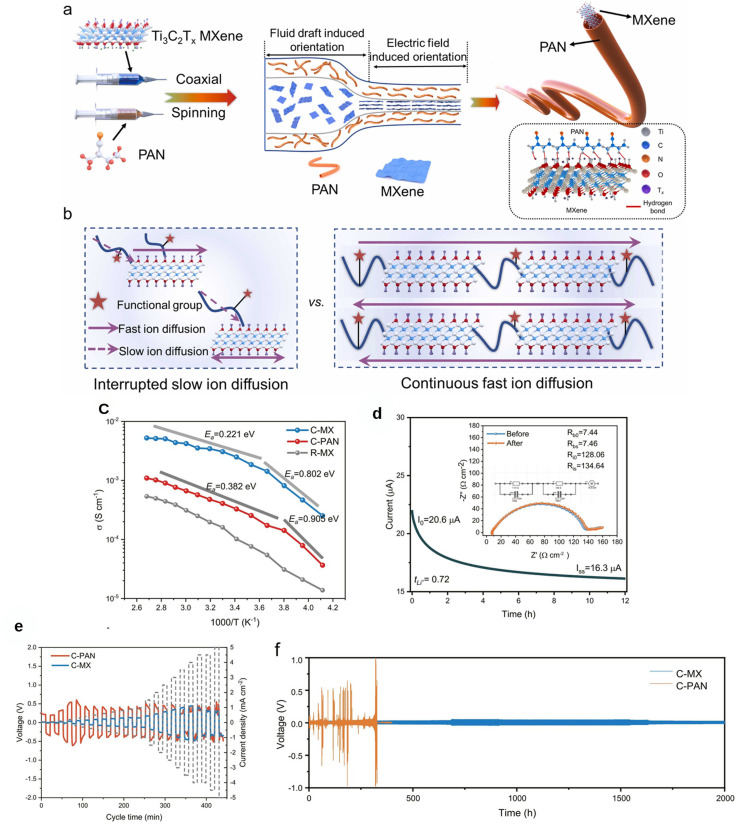
(a) Fabrication schematic-coaxial electrospinning of PAN with Ti_3_C_2_T_*x*_ confinement and field-induced orientation; (b) concept of interrupted slow *vs.* continuous fast Li^+^ diffusion under coaxial confinement; (c) Arrhenius conductivity showing *σ* ∼3.07 × 10^−3^ S cm^−1^ at 25 °C with reduced activation energy; (d) DC polarization/EIS giving Li^+^ transference number *t*^+^ ∼0.72; (e) critical-current-density test demonstrating high CCD under stepwise current; (f) long-term Li∥Li cycling at 1 mA cm^−2^ without shorting.^185^ This figure has been reproduced from ref. 185 with permission from Elsevier, copyright 2024.

Building on the same principles but further advancing the role of architecture, another study developed a three-dimensionally and coaxially MXene-confined solid polymer electrolyte (C-MX SPE), embedding Ti_3_C_2_T_*x*_ within electrospun PAN fibers to directionally accelerate Li^+^ transport.^185^ The C-MX SPE exhibited *σ*(25 °C) = 3.07 × 10^−3^ S cm^−1^ and *t*^+^(Li) = 0.72, which are markedly higher than those of a randomly dispersed MXene control (1.61 × 10^−4^ S cm^−1^; *t*^+^ = 0.22). Li‖SPE‖Li cells cycle stably for 2000 h at 1 mA cm^−2^, and flexible full cells deliver 101 mAh g^−1^ at 10C with 85.18% retention after 500 cycles. Crucially, these gains arise from the architecture, as shown in Fig. 13, as 3D/coaxial confinement lowers tortuosity and immobilizes anions along MXene/PAN interfaces rather than simply increasing MXene loading, thereby avoiding electronic percolation while improving interfacial contact. To enable rigorous cross-study comparisons, studies should standardize the *σ* measurement temperature at 25 °C (with Arrhenius/VTF analysis) and should use a standard film thickness and cell geometry to ensure that architecture-driven gains are not conflated with thickness/temperature effects.

Beyond its filler and interfacial regulator roles, MXenes can also serve as sacrificial/derivable scaffolds to construct ceramic-reinforced polymer electrolytes. By leveraging its 2D framework and surface chemistry, MXenes can template a stiff, percolating oxide network while retaining sufficient electronic/ionic pathways for stable plating/stripping. For instance, Liu *et al.*^149^ created an “MXene-derived TiO_2_” gel electrolyte for solid Zn-ion batteries by starting from a polymer/MXene matrix and inducing *in situ* oxidation of Ti_3_C_2_T_*x*_ into 2D TiO_2_ nanosheets within the polymer. This produced a robust hybrid network of amorphous TiO_2_ interwoven with residual MXenes throughout the gel, providing a high modulus and improved thermal stability while the remaining MXenes ensured adequate conductivity. The hybrid electrolyte enabled dendrite-free Zn plating even at ∼10 mA cm^−2^ and high areal capacities, far outperforming a polymer-only electrolyte that failed *via* Zn filament growth. Although originally demonstrated in Zn systems, the same sacrificial templating concept is transferable to Li through adaptation to non-aqueous chemistries and high-voltage cathodes, potentially reducing the ceramic loading required for reinforcement while maintaining processability. More recent analyses of solid-state MXene systems have pointed out that such MXene-derived oxide skeletons can also act as robust hosts for sulfide or garnet-type fillers, reducing the total ceramic loading needed to reach conductivities on the order of 10^−3^ S cm^−1^ and maintaining processability of the polymer phase.^186–188^ In this context, MXenes serve a dual role: initially as a conductive template guiding film formation and after partial oxidation, as a percolated ceramic backbone that mechanically reinforces the electrolyte and stabilizes the interfacial contact with Li or Zn metal anodes at high current densities.^186–188^

Overall, composite and hybrid electrolytes combining Ti_3_C_2_T_*x*_ with other phases are advancing performance to new heights. The role of MXenes can range from a conductive additive to a structural scaffold, an interfacial agent, or even a precursor to a ceramic. Researchers have developed mechanically tough, ionically fast, and electrochemically stable electrolytes by integrating MXenes. These properties are required for next-generation batteries such as all-solid-state Li metal batteries and flexible zinc batteries. One foreseeable future direction is to pair MXenes with sulfide or halide solid electrolytes (which have high conductivity but poor ductility) to form flexible composite membranes, building on preliminary studies that showed that MXenes can improve the processability of brittle sulfides.^79,87,189^ Another approach is the use of MXene heterostructures (*e.g.*, MXenes coated with another 2D materials such as h-BN or oxide nanosheets) to impart multiple functions (conductivity and stability).^32,190^ Finally, the modular nature of MXene chemistry (selectable metal cores and surface terminations) offers a rich design space for hybrid electrolyte engineering.^191^

Looking ahead to commercialization, several directions remain vital: (i) structure/chemistry optimization of MXenes (core carbide/nitride selection, interlayer spacing control, and surface termination engineering) and (ii) integration of MXene architectures with advanced solid electrolytes (*e.g.*, sulfides and halides) to couple interfacial compliance with wide electrochemical windows and high bulk conductivities. However, the utilization of this design space in practical devices will require parallel progress on the three fronts highlighted throughout this section: resolving the conductivity paradox by electronically insulating or architecturally confining MXenes inside electrolytes; replacing hazardous HF-based syntheses with scalable, fluoride-free routes that deliver comparable delamination and surface control; and standardizing electrochemical testing protocols so that claims of dendrite suppression and high-rate stability are validated under industrially relevant, lean-electrolyte conditions (Table 2).

**Table 2:** MXenes in electrolytes

MXene (form)	Electrolyte family	Cell/context	MXene's role	Electrochemical performance (capacity, mAh g^−1^)	Ref.
Ti_3_C_2_T_*x*_ (additive, dispersed)	Aqueous ZnSO_4_ (2 M)	Zn∥V_2_O_5_ full cell; Zn∥Zn symmetric	Interfacial mediator; Zn^2+^ flux homogenization; SEI stabilization	326.4 mAh g^−1^@1 A g^−1^ (initial); 192.9 mAh g^−1^ after 300 cycles; rate: 390.9 → 190.5 mAh g^−1^ (0.2 → 4 A g^−1^)	47
Ti_3_C_2_T_*x*_ in PVDF-HFP GPE with IL (EMIMBF_4_/Zn(OTF)_2_)	Ionic-liquid gel polymer	Na_3_V_2_(PO_4_)_3_∥Na full-cell demo; Zn∥Zn symmetric (separate)	Controls Zn^2+^ transport; suppresses parasitic reactions; smooth deposits	326.4 mAh g^−1^@1 A g^−1^ (initial); 192.9 mAh g^−1^ after 300 cycles; rate: 390.9 → 190.5 mAh g^−1^ (0.2→4 A g^−1^)	151
OH-functionalized Ti_3_C_2_T_*x*_ in PVA-KOH GPE	Alkaline gel polymer	Zn–air	Water-binding 3D network; zincophilic scaffold	*σ* ∼ 77.6 mS cm^−1^; stable cycling ∼160 h	143
Ti_3_C_2_T_*x*_-*g*-PMA in PVDF-HFP SPE	Solid polymer	Solid-state Zn	Reduced crystallinity; anion regulation; reinforced matrix	*σ*(25 °C) ∼ 2.69 × 10^−4^ S cm^−1^; Zn∥Zn > 1000 h	170
Ti_3_C_2_T_*x*_ in PEO-SN SPE	Solid polymer	Li metal	High *σ via* co-matrix + MXene; robust Li interface	151.7 mAh g^−1^; 99.3% retention after 300 cycles	172
				*σ*(35 °C) ∼ 2.17 × 10^−3^ S cm^−1^; long-term Li∥Li	
IL-grafted MXene (MXene-IL) in PEO SPE	Solid polymer	Li metal/LFP	Selective cation transport; widened ESW; passivated interphase	154.8 mAh g^−1^ over 120 cycles	97
ZIF-8@MXene in PEO composite	Polymer–ceramic composite	Li metal/LFP	“Bridge” between phases; fast-ion channels; flame retardance	*σ* ∼ 4.4 mS cm^−1^; *t*^+^ ∼ 0.76; Li∥Li ∼2000 h; LFP retention 89.6%/500 cycles	153
MXene/PAN nanofibers + LLZTO in PEO	Polymer–ceramic composite	Li metal	Compliant scaffold; improved contact; higher CCD	*σ* ∼ 10^−4^ S cm^−1^ (RT); tensile ∼2.37 MPa; extended life	152
3D/coaxially confined MXenes in PAN (C-MX SPE)	Architected SPE	Li metal/flexible	Directional Li^+^ highways; anion immobilization	*σ*(25 °C) 3.07 × 10^−3^ S cm^−1^; *t*^+^(Li) 0.72; Li∥Li 2000 h	185

## 5. Conclusions and outlook

In this review, we summarize the recent progress in MXene-based electrolytes and provide a detailed discussion of the various preparation strategies. Although direct HF etching is the most common method for the removal of Al layers from the Ti_3_AlC_2_ parent phase to produce Ti_3_C_2_T_*x*_ MXene, *in situ* HF generation is increasingly being encouraged owing to its advantages in reducing defects, controlling surface terminations, and improving the mechanical integrity of the resulting MXene. These factors directly affect the quality and electrochemical performance of MXene-based electrodes in battery applications.

Regarding intercalation strategies, Na^+^ and Li^+^ ions are often preferred over other cations (*e.g.* Mg^2+^ and K^+^) due to their favorable electric double-layer capacitance characteristics. Furthermore, the use of AI and machine-learning approaches is recommended to predict and evaluate the performance of Ti_3_C_2_T_*x*_-based electrodes intercalating multivalent ions (such as Ca^2+^ and Al^3+^), ionic species, or organic and inorganic molecules into the MXene structure.

These intercalated species act as effective interlayer spacers that help to prevent the restacking of the MXene flakes, thereby enhancing the electrochemical performance. In addition, the integration of MXene-based polysulfide adsorbents with electrochemically active components, such as Sb_2_O_3_, PPy, SnS_2_, and MnO_2_, warrants further investigation, because such hybrid systems have the potential to significantly improve energy storage capacity.

Although MXene-based electrolytes have demonstrated promising performance at the laboratory scale, achieving stable and reliable results at the pilot scale is essential for their integration into next-generation battery systems. Reaching this goal in a manufacturing environment requires systematic and stepwise progress with several critical aspects warranting further investigation.

(i) Performance evaluation of Ti_3_C_2_T_*x*_-based electrolytes: more comprehensive and systematic studies are needed to establish clear correlations between intrinsic material properties and electrochemical behavior. Particular attention should be paid to the electrochemical stability, physicochemical characteristics, and ionic conductivity.

(ii) Compatibility with other electrolyte systems: a thorough assessment of the interactions between Ti_3_C_2_T_*x*_-based electrolytes and other carbon-based or graphite-compatible electrolytes is necessary, including the potential detrimental effects of combining Ti_3_C_2_T_*x*_-based electrolytes with ether-based or conventional electrolyte formulations.

(iii) Safety considerations: both theoretical and experimental efforts must address safety challenges such as flammability, explosiveness, and volatility. Additionally, strategies to improve cost-effectiveness and optimize safety should be formulated to support the practical development of Ti_3_C_2_T_*x*_ MXene-based electrolytes.

Although multiple challenges must still be overcome to enable large-scale production and real-world integration, they can be addressed through sustained research and targeted engineering strategies. Thus, Ti_3_C_2_T_*x*_ MXenes are expected to remain highly promising candidates for the development of next-generation electrolyte technologies.

## Conflicts of interest

The authors declare that they have no competing financial interests or personal relationships that could have influenced the work reported in this study.

## Data Availability

No new data were created or analyzed in this study. All data supporting the findings of this work are available within the published literature cited throughout the manuscript.
